# Clades, classifications, and claims: evolution of organisms and their nomenclature in life science patents

**DOI:** 10.1093/jlb/lsaf026

**Published:** 2025-10-30

**Authors:** Nathan T Jacobs

**Affiliations:** Wolf, Greenfield & Sacks, P.C., Biotechnology Practice Group, 600 Atlantic Avenue, Boston, MA 02210, United States; Suffolk University Law School, Biotechnology Practice Group, 600 Atlantic Avenue, Boston, MA 02210, United States

**Keywords:** claim construction, enablement, evolution, microbiology, patents, written description

## Abstract

Organisms are complex biological entities that are less easily defined by specific structures or sequences than molecules, nucleic acids, and antibodies. Nor are they necessarily fixed in time or reproducible by repeatable methods, given their capacity for replication and mutation. Like organisms themselves, names and classifications also change over time as scientists better understand extant biodiversity. Taxonomy is the field of evolutionary biology concerned with classifying, naming, and identifying organisms, while phylogenetics concerns organisms’ evolutionary history and relatedness. Here, I review the challenges evolution poses for patentees, using the examples of evolving influenza viruses and bacterial classifications, and Federal Circuit decisions relevant each issue. I conclude that careful consideration of organisms’ evolutionary histories and the systematics underlying their classification in specification drafting allows patentees to: (i) mitigate the impact of scientific disagreement (such as the ‘species problem’ in microbiology) in claim construction; (ii) limit the effects of changing classifications on infringement analysis; (iii) describe generic categories of related organisms to encompass later-arising ones; and (iv) bolster compliance with the written description and enablement requirements of 35 U.S.C. § 112(a). Accordingly, patentees might address the challenges that evolutionary uncertainty poses for organism-centered patents by embracing these areas of evolutionary biology.

## I. INTRODUCTION

The evolutionary history and relatedness of organisms are central to the working of many inventions in biotechnology. Prophylactic vaccines, for example, use pathogens or their components to induce adaptive immune responses, thereby reducing the risk of infection by closely related pathogens. The breadth of protection afforded by a particular vaccine component is pathogen-dependent—the same measles vaccine, containing a single strain of measles virus, has been in use for over 50 years,^1^ whereas seasonal influenza vaccines contain components from multiple viruses of different lineages and are updated regularly to account for viral evolution.^2^ Other inventions, such as live biotherapeutic products, direct-fed microbials, and postbiotics, harness utility of microorganisms and metabolites they produce through fermentation.^3^ Alternatively, organisms may be genetically modified to provide new utility, such as pest resistance or nutritional quality in agriculture.^4^ Where these utilities are observed in one organism, the question arises of which closely related organisms provide similar utility, or which related organisms may be engineered to achieve similar benefits, respectively. Addressing these questions of conserved properties between organisms is critical for patentees’ compliance with the written description and enablement requirements for patents on inventions involving organisms.^5^

Complicating the issue is the difficulty in defining and reproducing organisms as physical entities, in contrast to small molecules or proteins such as antibodies. Patents are written documents, ending in claims defining what the inventor regards as the invention.^6^ In the small molecule context, a patent’s claims may use graphic formulae and chemical nomenclature to identify the compound(s) invented.^7^ The *Papesch* court recognized, however, that the *things* patented are the compounds so identified, not the formulae and nomenclature themselves.^8^ While such representations may suffice for small molecules, organisms (and their cells) are characterized by their ability to replicate, with changes accumulating over successive generations. The influenza A/(H3N2) virus subtype, for example, encompasses influenza A viruses that may have been isolated nearly 60 years apart but are nonetheless related by descent and genetically closer to each other than to viruses of the influenza A/(H1N1) virus subtype.^9^ Cell lines, as another example, are often referred to by a shorthand name such as MDCK or HEK293, reflecting their original isolation—Madin and Darby’s isolation of cells from a canine kidney (MDCK),^10^ or a human embryonic kidney cell line designated 293 (HEK293).^11^ But through over 50 years of passaging since their original isolations, substantial diversity has accumulated among lineages that authors may still call ‘MDCK’ or ‘HEK293’.^12^ Biological nomenclature, then, may encompass diverse categories of related organisms or biological entities, more broadly than chemical nomenclature or formulae identify a specific compound.

The difficulty in defining an organism by name alone may be addressed by depositing the organism itself, making it available to the public to satisfy the requirements of 35 U.S.C. § 112.^13^ But making such deposits poses at least three issues for patentees. First, patents directed to bacteria and their products often recite deposited strains in the claims.^14^ MacLeod identifies several judgments from foreign courts that suggest these claims are broader in practice than they might appear, but also recognizes the other potential interpretation of these claims as extremely narrow, extending only to the deposited strain’s descendants.^15^ Second, making a deposit during prosecution, such as to address an enablement rejection, may unintentionally limit claim scope to require the deposited material even if the deposited material is not recited.^16^ Third, where multiple deposits are made, they may be perceived as collectively representing a claim term, with the commonalities among them being construed as claim limitations even if a broader scope would still be distinct from the prior art.^17^  Sections III.A and III.B discuss these second and third issues in more detail, respectively.

The potential limitations or uncertainty posed by deposits are especially undesirable where the specific organisms used to exemplify an invention are not truly necessary to implement it, or even commercially relevant over the 20 years of a patent’s term. Efforts to stabilize influenza virus antigens, for example, often target residues that are conserved across a diversity of subtypes, let alone between more closely related viruses within a subtype.^18^ Public health authorities regularly update the candidate viruses for inclusion in seasonal influenza vaccines to account for viral evolution,^19^ making it foreseeable that these modifications may be applied to proteins of later-arising viruses while the exemplified viruses become outdated. Regulatory considerations, too, may warrant replacing an exemplified organism with another in a commercial product, such as genetic stability in the context of a live biotherapeutic product.^20^ Claims limited to specific organisms, then, are not likely to fully serve the needs of patentees.

Genus claims, encompassing whole categories of organisms, pose their own challenges with respect to 35 U.S.C. § 112(a). The species concept is controversial in bacteriology and virology,^21^ introducing uncertainty into the meaning of claims referring to bacteria and viruses by a categorical name such as that of a species. Nomenclature for these organisms also changes frequently as knowledge of biodiversity,^22^ and the organisms themselves,^23^ evolve, but the prohibition against added matter can cause a mismatch between terminology and knowledge reflected in a specification filed today and that in use 15 years later when infringement is accused.^24^ And as others have articulated, genus claims have received increasingly negative treatment with respect to the written description and enablement requirements.^25^ The Federal Circuit’s shift toward disfavor of genus claims is reviewed by Karshtedt *et al.* in *The Death of the Genus Claim*.^26^ Lemley & Sherkow review the implications of this ‘war on genus claims’ in the specific context of antibodies in *The Antibody Patent Paradox*, noting an apparent primacy of structure for compliance with both doctrines.^27^ The structural features defining a general category of organisms are less immediately apparent, however, than for chemical compounds or antibodies. And, in contrast to small molecules or proteins that can be produced by repeatable methods, the tendency of organisms to change over successive generations complicates attempts to define them by a specific sequence, as may be done for antibodies.

The problems evolution poses for organism-centered claims may be addressed by systematics, the branch of evolutionary biology concerned with classification of organisms, accounting for the evolutionary processes that produce observed diversity. Within systematics, taxonomy is the study of classification and nomenclature, while phylogenetics is the study of evolutionary history using genetics.^28^ Here, I review issues raised by the evolution of bacteria and influenza viruses, the Federal Circuit’s treatment of similar issues and potential solutions grounded in the systematics of these organisms. Bacteria are particularly illustrative of issues arising from changes to classification schemes and nomenclature, while influenza viruses highlight issues arising from rapid evolution during a 20-year patent term. The issues are not mutually exclusive, though, as viral taxonomy has recently undergone a substantial albeit controversial shift,^29^ and bacterial populations change over time in response to vaccine-related selection.^30^

I focus the discussion on bacteria and influenza viruses as examples from my scientific research experience, but eukaryotes are not immune to these issues. Advances in phylogenetic understanding have also warranted reclassifications in medically relevant fungi^31^ and mosquitoes^32^ and agriculturally relevant plants.^33^ Human activity can also drive animal, plant, and fungal evolution over short time scales, such as through urbanization,^34^ agriculture,^35^ and climate change.^36^ And while reclassifications are perhaps not as frequent as in bacteria or evolution not quite as rapid as in seasonal influenza viruses, multiple written description and enablement cases discussed below concerned diversity and conservation of features within eukaryotic taxa.

This is not to say that the issues are precisely analogous to eukaryotes—the ‘species problem’ for bacteria and viruses stems partly from difficulty applying workable eukaryotic species concepts to other domains of life.^37^ These concepts, then, may instead be more applicable at taxonomic scales below the species level, such as plant cultivars^38^ or animal genetic backgrounds or breeds.^39^ Beyond whole organisms, the HEK293 and MDCK cell examples above illustrate that a cell population’s name may encompass more diversity than initially apparent and that non-trivial changes can arise in cultured eukaryotic cell populations over time.^40^ Systematic concepts have also been applied to better understand and classify somatic cell types, given their ability to change and diverge with replication. The cluster differentiation system, for example, seeks to provide a uniform nomenclature for cell surface markers based on binding by specific antibodies,^41^ allowing cells to be classified by the presence or absence of certain markers.^42^ Phylogenetics, too, has been used to analyze evolution of cell populations within organisms, such as evaluating B-cell lineages to identify mutations important for antigen binding^43^ and stratifying cancers into subtypes by inferring their evolutionary trajectories.^44^ The details will depend on the context of an invention, but applicants working with replicating entities should find some benefit in proactively addressing questions of diversity within a category, how that diversity or understanding of it might change over time, and biologically relevant features conserved within the category.

## II. BACTERIAL AND VIRAL SYSTEMATICS

This section provides an overview of bacterial and viral taxonomy and phylogenetics as relevant to the discussion in Section III. Section II.A summarizes relevant systematics terminology. Section II.B describes the progression of bacterial classification schemes from 1923 to present, while Section II.C describes the classification of evolving influenza viruses.

### II.A. Taxonomy and Phylogenetics Vocabulary

Systematics is concerned with the diversity of organisms and the relationships between them, and includes taxonomy and phylogenetics.^45^ Taxonomy is concerned with classification, nomenclature, and identification of organisms.^46^ Classification is the ordered arrangement of organisms into groups (taxa) such as species and genera.^47^ Nomenclature is the labeling of taxonomic units so defined, as opposed to classification itself.^48^ Identification is the assignment of a given organism to a taxon based on its observable features.^49^ While classifications may be grounded in various observable criteria, they will ideally reflect phylogenetic relationships.^50^

Phylogenetics is concerned with the evolutionary history of organisms and their relatedness by descent.^51^ The evolutionary history of a group of organisms may be referred to as its phylogeny, which may be represented by a phylogenetic tree showing the inferred order of divergence from common ancestors.^52^ A clade is a monophyletic group, one that includes all descendants of a common ancestor.^53^ A sister clade is the closest clade to another clade in a phylogenetic tree, such that two sister clades and their most recent common ancestor form a monophyletic group.^54^ Among non-monophyletic groups, paraphyletic ones include some but not all descendants of a common ancestor (eg ‘reptiles’, including lizards, turtles, and crocodiles but excluding birds, which are a sister clade to crocodiles).^55^ Polyphyletic groups, as another non-monophyletic example, do not include the common ancestor of all members (eg ‘vultures’, including New World vultures but not their sister clade of storks, and including Old World vultures but not their sister clade of birds of prey).^56^

### II.B. History of Bacterial Classification

Over the past century, bacterial systematics has progressed from classifying bacteria according to their phenotypes (eg shape, motility, behavior in culture), then to using molecular methods such as DNA hybridization or 16S ribosomal RNA (rRNA) gene sequence similarity, and most recently to a comprehensive genome-based taxonomy based on sequences of full genomes and the proteins they encode.^57^ Since its first publication in 1923, Bergey’s Manual of Determinative Bacteriology has been a foundational resource for bacteriologists to identify a bacterial isolate as belonging to a given group based on its observable characteristics.^58^ Bacteria of the genus *Escherichia*, for example, are described as Gram-negative, straight, cylindrical rods, with certain patterns of motility, oxygen tolerance, fermentation of certain carbohydrates, growth on certain substrates, and lack of hydrogen sulfide production.^59^

With advancements in molecular methods in the mid-to-late 20th century, molecular genomic and genetic features were incorporated into classification schemes.^60^ In 1987, an ad hoc committee on reconciliation of approaches to bacterial systematics proposed that a phylogenetic species definition would include strains with approximately 70% or greater DNA–DNA relatedness and with 5 °C or less Tm.^61^ The sequence encoding RNA of the small ribosomal subunit (16S rRNA, in bacteria) mutates slowly over time, permitting detection of relatedness.^62^ In 1994, Stackebrandt & Goebel noted that the relationship between DNA similarity and 16S rRNA homology was non-linear but that species having 70% or greater DNA similarity usually have more than 97% sequence identity between 16S rRNA gene sequences.^63^ Accordingly, laborious DNA:DNA reassociation experiments could be avoided for bacteria sharing less than 97% identity between 16S rRNA gene sequences.^64^ In 2006, Stackebrandt & Ebers recommended updating this conservative threshold to 98.7–99%, after re-evaluating the correlation with more recent data.^65^ In each case, this threshold provided a benchmark for identifying bacteria as belonging to different species, if relatedness between 16S rRNA gene sequences fell short.^66^ Because the relationship between genomic identity and 16S rRNA gene sequence identity is non-linear, however, bacteria may share at least 97% or at least 99% identity between their 16S rRNA gene sequences but still belong to different species.^67^ These DNA and 16S rRNA-based molecular methods strengthened taxonomy by incorporating phylogenetic information, then, but were not without limitation.^68^

As more bacterial genome sequences became available, Konstantinidis & Tiedje advanced the metric of average nucleotide identity (ANI).^69^ ANI is calculated by comparing genomes to identify portions conserved between them, excluding genome-specific portions, and calculating the average nucleotide identity between all conserved portions.^70^ Using this metric, Konstantinidis & Tiedje found that 94% ANI corresponded to the traditional 70% DNA–DNA reassociation standard.^71^ Shortly afterward, Goris *et al.* proposed a 95% ANI cutoff for demarcating species.^72^ A high-throughput comparison of each pair of 90,000 genomes then revealed a discontinuous distribution, with ANI values generally being 95% or above or less than 83%.^73^ While the extent and meaning of this discontinuity have been controversial,^74^ it remains observed in nature, likely as a result of multiple combined ecological and evolutionary forces.^75^

This 95% ANI circumscription radius formed the basis of Parks *et al.*’s species cluster definition in the publication of the Genome Taxonomy Database (GTDB).^76^ In this comprehensive genome-based taxonomy, a species cluster is defined by a reference genome and ANI circumscription radius, encompassing all genomes falling within that radius from the representative genome.^77^ For consistency with historic taxonomy, the representative genome is typically the type strain of a corresponding named species, and the ANI circumscription radius is typically (i) 95%, or (ii) if two representative genomes are within 95% ANI, the distance between the two representative genomes to maintain separation of the two species clusters.^78^

Over time, each of these classification schemes has appeared in US patents and applications related to bacteria. The *Chakrabarty* patent,^79^ for instance, cites to Stanier *et al.*’s phenotypic characterization of aerobic bacteria in the genus *Pseudomonas*.^80^ Subsequent patents and applications recite DNA:DNA hybridization,^81^ 16S rRNA gene sequences and 97% identity thereto,^82^^,^^83^ 95% ANI to a deposited bacterium’s genome,^84^ and GTDB classifications.^85^

### II.C. Classification of Influenza Viruses

van Regenmortel wrote repeatedly, both alone and with colleagues, on the species problem in virology.^86^ The ecology and evolution of influenza viruses specifically is reviewed by Wille and Holmes,^87^ with an overview of the relevant features summarized here. The family Orthomyxoviridae includes four species, influenza A virus, influenza B virus, influenza C virus, and influenza D virus.^88^ Of these species, seasonal epidemics in humans are caused by influenza A viruses (IAVs) and influenza B viruses (IBVs).^89^ The genomes of IAVs and IBVs are divided into eight RNA segments, ordered by length, with segments 4 and 6 encoding hemagglutinin (HA) and neuraminidase (NA) viral surface proteins, respectively.^90^ IAVs are divided into subtypes based on their HA and NA proteins, of which 18 HA subtypes and 11 NA subtypes are recognized.^91^ Of these subtypes, H1, H2, H5, H6, H8, H9, H11, H12, H13, H16, H17, and H18 form the Group 1 HA clade, while H3, H4, H7, H10, H14, and H15 form the Group 2 HA clade, with the combination of H2 and H5 HAs being the sister clade to H1 HAs, and the set of H4 and H14 HAs being the sister clade to H3 HAs.^92^ IBVs are not divided into subtypes as IAVs are but rather into two distinct lineages, a B/Victoria lineage and B/Yamagata lineage, based on similarity to influenza B/Victoria/2/87 and B/Yamagata/16/88 viruses, respectively.^93^ Individual virus isolates are characterized according to these subdivisions, with an isolate being identified by a species (eg A or B), location of isolation, year of isolation, serial number among isolates from that location in the year, and subtype or lineage, as appropriate for the species.^94^ The isolate A/Panama/2007/1999(H3N2), for example, was an influenza A virus isolated in Panama in 1999, being the 2007th IAV isolated from Panama that year and having HA and NA proteins belonging to the H3 HA and N2 NA subtypes.^95^

Influenza viruses evolve rapidly, allowing evasion of prior immunity and causing seasonal epidemics.^96^ Smith *et al.* analyzed the antigenic evolution within the A/(H3N2) subtype, finding that viruses isolated after 1968 exhibited increasing genetic and antigenic distance from the Hong Kong 1968 antigenic cluster with time.^97^ Exploring this historic diversity, Huber *et al.* reported that a given IAV H3 HA antigen elicited responses that neutralized only viruses closely related in time and so used a multivalent vaccine encoding multiple historic H3 HA antigens to elicit broad immunity within the subtype.^98^

Beyond these antigenic changes over time, subtypes may also contain multiple antigenically distinct clusters circulating at any one time. To reflect this antigenic diversity, subtypes and lineages may be divided into clades, identified by amino acid changes that distinguish them from parental viruses.^99^ The names of these clades and subclades are nested—the A/(H3N2) subtype contains clade 3C, having subclades 3C.1, 3C.2, and 3C.3, with 3C.2 having subclades 3C.2a and 3C.2b, and 3C.2a having the subclade 3C.2a1.^100^

## III. EVOLUTION IN TENSION WITH 35 U.S.C. § 112

This section proceeds in four subsections. Section III.A relates to the controversy surrounding the ‘species problem’ in bacteria and viruses and different species concepts in the scientific literature, insofar as this controversy may affect claim construction. Section III.B relates to changes in nomenclature that may occur during a patent’s term, causing a mismatch between a patent’s terminology and the terminology in use at the time of accused infringement. Section III.C relates to changing implications of nomenclature as biology advances and populations evolve over a patent’s term, as they relate to enablement and written description. Section III.D relates to the diversity across taxonomic ranks and the relationship of that diversity to written description and enablement. Each section is divided into subsections, first reviewing the problem as it arises in the scientific literature, followed by case law relevant to the issue, and concluding with options for applicants to mitigate the issue in specification drafting.

### III.A. The ‘Species Problem’ as Discussed in the Scientific Literature May be Less Relevant to the Context of a Patent Application

#### III.A.1. Species Concepts and Definitions are Controversial in Scientific Literature

The concept and definition of a ‘species’ have been continuously debated in bacteriology and virology. This is unsurprising given the age of the species concept—recognizing groups of organisms by resemblances between the ‘shapes of their parts, or of their whole body’ can be traced to Aristotle,^101^ and even Linnaean taxonomy dates to the 18th century.^102^ While bacterial and viral species concepts and definitions have been revised over time,^103^ the only certainty one can draw from the history of this debate is that no universal definition of a species is recognized in either field.

Controversy in the species concept as applied to bacteria and viruses is at least partly related to difficulty of applying definitions originally developed for eukaryotes. The biological species concept, as initially described, considered species ‘as groups of actually or potentially interbreeding natural populations which are reproductively isolated from other such groups’.^104^ While this concept was workable for animals, it is challenging to apply to bacteria, which are single-celled and reproduce by binary division, rather than sexual reproduction involving meiosis and fertilization.^105^ Even horizontal gene transfer, which may be analogized to prokaryotic sex, can involve acquisition of genes from very divergent sources, and so discrimination based on interbreeding is less applicable to prokaryotes.^106^ This tendency to exchange genetic material also blurs the division between closely related species, with species boundaries between certain recombining bacteria being considered ‘fuzzy’.^107^

Virologists, too, have wrestled with the limited applicability of Mayr’s original biological species concept to viruses.^108^ Whereas horizontal gene transfer occurs between even distantly related bacteria recognized as different species, genetic exchange is constrained between even influenza A viruses recognized as belonging to a single species.^109^ Division of the genome into eight distinct RNA segments allows mixing of segments within co-infected cells to produce virions with genome segments from different parental viruses, with this process of reassortment being analogized to sexual reproduction in segmented viruses.^110^ While reassortment happens readily between closely related viruses, such as those replicating during a single infection,^111^ reassortment between IAVs of different subtypes is constrained by incompatibility between genome segments in packaging into virus particles^112^ or between proteins encoded by genome segments from divergent virus backgrounds.^113^

Nonetheless, researchers recognize that a ‘species’ definition is useful insofar as it circumscribes a group of organisms sharing a certain (high) degree of commonality. With respect to bacteria, an ad hoc committee for re-evaluation of the species definition in bacteriology recommended defining a species pragmatically, as ‘a category that circumscribes a (preferably) genomically coherent group of individual isolates/strains sharing a high degree of similarity in (many) independent features, comparatively tested under highly standardized conditions’.^114^ Following this trend, Doolittle and Papke articulated the need for *some* working definition in bacteriology: ‘What we want from a species definition is a set of easily applied and stable rules by which to decide when two organisms are similar enough in their genomic and/or phenotypic properties to be given the same name’.^115^ In publishing the Genome Taxonomy Database, Parks *et al.* proposed a quantitative species definition based on genomic similarity, in which a ‘species cluster’ encompasses every genome within a fixed ANI distance (often 95%) to a designated representative genome (often of a type strain).^116^ The same research group subsequently noted this was a pragmatic approach to characterizing biodiversity rather than accepting the limitations from leaving it unclassified, citing Doolittle & Papke’s desire for a pragmatic definition.^117^ In virology, van Regenmortel & Mahy similarly articulated the practical purpose of ‘partition[ing] the world of viruses into a coherent scheme of easily recognizable entities that answers to the everyday needs of practicing virologists’.^118^ Given the diversity in viral genome architectures and replication programs, different viral taxa will have different criteria for demarcation but still aim to guide the decision of whether a newly isolated virus belongs to an existing species or warrants creation of a new species.^119^

To an inventor or patentee, the relevant commonality and applicable definition of a category of organisms will depend on the context of the invention. The *Chakrabarty* patent, for instance, concerned bacteria of the genus *Pseudomonas* modified by introduction of plasmids to make them capable of digesting oil.^120^ Live biotherapeutic products, as another example, can benefit hosts by inhibiting the growth of pathogenic organisms or limiting inflammation through interaction with the mucosal immune system.^121^ Postbiotics, containing bacterial-derived metabolites, have related potential to influence human metabolism.^122^ The skilled bacteriologist, then, may be concerned with the depth of conservation of the relevant metabolic pathways, interactions with a host or other colonizing bacteria, or ability to be modified for a particular purpose.

The skilled virologist seeking an effective vaccine, on the other hand, may be concerned with the subtype or antigenic clade of an influenza virus. Seasonal influenza vaccines typically target IAVs of both the A/(H1N1)pdm09 and A/(H3N2) subtypes, as well as IBVs of the B/Victoria lineage.^123^ The generic term ‘influenza virus’, or even species names ‘influenza A virus’ and ‘influenza B virus’, may not be sufficient to address the intraspecies diversity relevant to vaccine or other antiviral development. Or, those seeking to produce a defective virus that interferes with one causing an active infection may be concerned with the ability of defective genome segments to be packaged into virus particles with those of the infecting virus being targeted.^124^ In both contexts, inventors may appreciate the issues surrounding the species concept but may not require a universally accepted definition to comply with the requirements for patentability. Analogous concerns may apply to inventors in other fields facing disagreements of classifying other relevant populations, such as hematologic malignancies and B- or T-cell populations.^125^

#### III.A.2. The Intrinsic Record, Reflecting the Patentee’s Intent, is Controlling in Claim Construction

The intrinsic record reflects a patentee’s provision to the public of notice of a patent’s claim scope.^126^ The Federal Circuit in *Immunex* explained this principle by noting that the drafter is in the best position to anticipate and address ambiguity, because they write the claims, prepare the specification, and respond to Office Actions.^127^ There, the court rejected an appellant’s argument that the Board did not adequately consult extrinsic evidence to determine whether the term ‘human antibody’ had an established meaning in the art, independent of the specification.^128^ Even if extrinsic evidence is useful in resolving ambiguity, the court declined to allow a litigant to introduce extrinsic evidence that disregards the language of the patent itself.^129^

While *Immunex* applied the ‘broadest reasonable interpretation’ standard used in prosecution, claim construction in *inter partes* review and in district courts follows the *Phillips* standard:^130^ claim terms are given their plain and ordinary meaning to the skilled artisan after reading the entire patent.^131^ In *Phillips*, the *en banc* Federal Circuit clarified the relative weight of different forms of intrinsic and extrinsic evidence.^132^ Within the intrinsic record, terms’ use in the context of the claims is highly instructive.^133^ The court recognized, however, that claims do not stand alone but are part of ‘a fully integrated written instrument’, appearing at the end of the specification.^134^ When considering a claim term in context, then, the specification is the ‘single best guide to the meaning of a disputed term’.^135^ Consistent with this principle, the specification may provide a special definition different from the ordinary meaning or intentionally disavow a certain scope.^136^ In both cases, the inventor’s intent expressed in the specification controls.^137^ While the prosecution history is also intrinsic evidence, the court noted that it represents an ongoing negotiation with the Patent Office and often lacks the clarity of the specification, making it less useful in construction.^138^ Finally, the court authorized use of extrinsic evidence such as dictionaries and expert testimony, but emphasized the biases and lower value of such evidence compared to the intrinsic record.^139^

In keeping with *Phillips*, if some ambiguity remains after consulting the specification, an applicant’s treatment of claim terms during examination may impart meaning to an otherwise undefined term or narrow one with a broader ordinary meaning.^140^ The *Sunovion* court, for example, considered construction of the term ‘essentially free’ as containing less than 0.25% of a levorotatory isomer.^141^ While the term appeared only in the claims and was not defined, the patentee had repeatedly referred to a working example, describing a pure form of a dextrorotatory isomer as having less than 0.25% of the levorotatory isomer, to support patentability.^142^ Accordingly, the patentee’s repeated and definitive reference to this attribute in prosecution gave meaning to the otherwise undefined term.^143^

Actions in examination other than argument may also affect claim construction. In *Genzyme*, a patentee had made a biological deposit of a vector to overcome an enablement rejection related to claims reciting cells for producing an enzyme, transformed with a recombinant vector encoding α–Gal A.^144^ Because the examiner considered the deposit ‘essential’ to the invention and the patentee had deposited it without argument, the court affirmed a construction that required the deposited vector.^145^ Judge Linn, in dissent, criticized this view in part because the term ‘recombinant vector’ had been removed from the claims by a later amendment.^146^ The majority, however, considered that in view of the Office’s regulations for amendments after final rejection and the patentee’s lack of formal comment, this amendment could not have been accepted if it had actually broadened the claims.^147^

If the intrinsic record does not resolve ambiguity in a term, other sources in the art will affect construction, as *Phillips* permits using extrinsic evidence in claim construction (keeping in mind its flaws).^148^ In *MIT*, for example, the Federal Circuit considered that where the term ‘scanner’ was not defined explicitly or implicitly, and the specification disclosed only one type of scanner, it was appropriate to consult dictionaries and, if the dictionaries were not helpful, other sources in the art.^149^ On these grounds, the court affirmed the district court’s construction adverse to the patentee, that the term ‘scanner’ (i) based on dictionary definitions, required relative movement between a scanning element and the object being scanned, excluding flying spot scanners and (ii) based on expert testimony and technical articles, required close proximity between the scanner and the object being scanned.^150^ The court’s analysis of the ‘close proximity’ requirement also considered the meaning at the time of filing, as discussed below in Section III.B.

#### III.A.3. Applicants May Provide a Controlling Definition of an Organismal Category Based on the Literature Relevant to the Invention’s Context

To reduce uncertainty in claim construction concerning organismal classifications (taxa), applicants may consider providing an express definition grounded in the relevant literature, depending on the intended meaning of a taxon in the context of the invention. Just as Doolittle & Papke articulate the desire for a set of easily applied rules by which to decide two organisms are similar enough to be given the same name,^151^ a patentee may desire a set of easily applied criteria to determine whether another organism meets the requirements of a claim. While researchers may debate varying species concepts grounded in different aspects of biology, different biological underpinnings and their associated species definitions may be more or less relevant to a given invention. Where bacteria are concerned, if there is a consensus that taxonomic information is contained within the genome,^152^ the relevant definition may be a genomic one, for example. On the other hand, where influenza viruses are divided into phylogenetic subgroupings that vary in their antigenicity and structure of their HA and NA proteins,^153^ the relevant definition may be based on phylogeny of genome segments encoding HA and NA proteins. Inventors in other fields may similarly consider controlling definitions to mitigate the effects of disagreement in classification schemes for other populations (eg hematologic malignancies or immune cell types).^154^

To suggest that an applicant describe what is claimed and define key terms seems, admittedly, obvious. Because a specification need not disclose what is well known in the art,^155^ though, applicants may be tempted not to define terms that could be considered well known, to avoid being held to an unintentionally limiting definition. Without a controlling definition in the intrinsic record, however, extrinsic evidence such as disagreement in the evolutionary biology literature concerning the species concept and whether species definitions are even possible (or analogous disagreements in other fields) may introduce uncertainty into claim construction. On balance, an inventor with more knowledge of the organismal biology relevant to their invention may mitigate this uncertainty by clarifying their intended meaning in the specification.

Beyond providing more control in claim interpretation, a carefully considered intention and controlling definition would provide additional support for written description and enablement over the scope of relevant taxa, discussed further in Sections III.C and III.D. The temptation to avoid defining terms well known in the art is particularly relevant to organisms that can be identified as belonging to already known taxa, as their taxonomic identity alone is unlikely to provide novelty. The patents at issue in *Chakrabarty*, *BASF*, and *Vaeck*, for example, concerned genetically modified forms of *Pseudomonas* bacteria,^156^  *Brassica napus* plants and cells,^157^ and Cyanobacteria,^158^ organisms whose naturally occurring forms were known to exist before the patents’ respective filing dates. As the Federal Circuit clarified in *Juno*, however, the written description requirement applies to the entirety of a claim, including features that are not a point of novelty.^159^ And with respect to enablement, the court has agreed with the notion that if something is so well known, then there should be no trouble in documenting it.^160^ Providing such documentation would not only mitigate the influence of extrinsic evidence but also support analysis of accused infringement at later parts of a patent’s term, when organismal biology may have advanced substantially since the application’s filing date, as discussed in Section III.B below.

### III.B. Nomenclature at a Patent Application’s Filing Date May Become Outdated over the 20 Years of a Patent’s Term

#### III.B.1. Bacterial Nomenclature Evolves with Increasing Knowledge of Bacteriology

A tangible organism that is unchanged, at least to the extent possible for a preserved bacterium, may be encompassed by multiple distinct classifications in a 20-year patent term. As one example, the bacterial strain H2_18 deposited as DSM 108153/JCM 34816 was isolated in 2014 and deemed an ‘uncultured *Clostridium* sp. isolate’ when its sequenced genome was published in 2016.^161^ Successive releases of the Genome Taxonomy Database identified the strain’s genome as belonging to an unnamed species cluster within the genus *Faecalicatena* (Release 89 on June 17, 2019) and then to the species cluster *Mediterraneibacter massiliensis* (Release 95 on July 17, 2020).^162^ A 2021 analysis of this and other genomes classified it in a distinct species within the genus *Faecalicatena*, with the proposed species *Faecalicatena acetigenes* having H2_18 as its type strain.^163^  *Faecalicatena acetigenes* is not recognized as a species cluster in the Genome Taxonomy Database, however, given placement of the H2_18 strain’s genome in the previously established *M. massiliensis* cluster.^164^ Starting from 2014, then, strain H2_18 could be identified as *Clostridium* sp. (2014–19), *Faecalicatena sp001487105* (2019–20), *M. massiliensis* (2020–21), and either *F. acetigenes* or *M. massiliensis* depending on the underlying classification framework (2021–25).

These shifts in nomenclature and reclassifications have the potential to change the actual scope of organisms encompassed by a given name. This issue is particularly pronounced where organisms in one taxon are separated into multiple others—beyond the specific example of H2_18 above, many genomes originally grouped in the genus *Faecalicatena* have since been reassigned to other genera, including *Mediterraneibacter* and *Muricomes*.^165^ Conversely, effective publication of the genus *Mediterraneibacter* united multiple species belonging to distinct genera (*Ruminococcus faecis*, *R. lactaris*, *R. torques*, *R. gnavus,* and *Clostridium glycyrrhizinilyticum*) within this new genus, together with the newly published species *M. massiliensis*.^166^

Beyond individual strains or species, whole genera have been revised over time. *Clostridium*, as a named genus, was long considered a ‘dumping ground’ for spore-forming anaerobes,^167^ the only additional criterion for admission being that they do not reduce sulfate (or would be classified as *Desulfotomaculum*).^168^ In the same issue of the *International Journal of Systematic and Evolutionary Microbiology* where Stackebrandt & Goebel highlighted the relationship of 16S rRNA gene sequence similarity to DNA:DNA reassociation, Collins *et al.* analyzed the phylogenetic relationships between bacteria in this diverse genus by comparing their 16S rRNA gene sequences.^169^ This analysis revealed many distinct clusters, numbered I through XIX, each being defined by the same minimum depth on a phylogenetic tree (ie representing a similar minimum divergence from the most recent common ancestor to all clusters).^170^ Subsequent publications of new *Clostridium* species would then typically identify the phylogenetic cluster to which the species belonged.^171^ The genus *Bacillus*, including *aerobic* spore-formers, was similarly recognized as non-monophyletic, with an analogous phylogeny of 16S rRNA gene sequences revealing at least four distinct clusters (eg a ‘*Bacillus subtilis* cluster’, including *B. subtilis* and *B. stearothermophilus*).^172^ In more recent genome-based taxonomy, both *Clostridium* and *Bacillus* were divided into many distinct genera and families (121 separate genera across 29 different families for *Clostridium*, and 81 genera across 25 families for *Bacillus*) to resolve these polyphylies.^173^ As a result, the Genome Taxonomy Database includes many *Clostridium* and *Bacillus* genus names with alphabetical suffixes (eg *Clostridium_F*, *Bacillus_B*), reflecting the polyphyletic nature of these genera as previously named.^174^

In other instances, classifications may be retained even where they are recognized as being erroneous or inconsistent with general classification frameworks. Bacteria causing shigellosis (dysentery) were historically classified as *Shigella flexneri*, *S. sonnei*, *S. boydii*, and *S. dysenteriae*, in a distinct genus from *Escherichia coli*.^175^ Accumulating evidence suggests that these bacteria are genomically very similar to *E. coli*, with virulence resulting from acquisition of transferable genetic elements.^176^ Given this close genomic similarity, recent Genome Taxonomy Database releases assign *Shigella* strains to the single species *E. coli*.^177^ Despite the recognition that *Shigella* strains are genomically coherent with *E. coli*, however, the four *Shigella* species names remain in use due to their clinical significance and to avoid confusion in medical microbiology.^178^

#### III.B.2. Influenza Virus Nomenclature Evolves to Account for Antigenic Shift and Antigenic Drift

Subtypes of ‘seasonal influenza viruses’ have changed multiple times since the 1918 pandemic.^179^ The predominant subtype causing seasonal epidemics was A/(H1N1) following the 1918 pandemic, until it was replaced by A/(H2N2) in 1957, which was, in turn, replaced by A/(H3N2) in 1968.^180^ Then, since 1977, influenza A/(H1N1) and A/(H3N2) viruses have cocirculated seasonally in humans.^181^

The 2009 influenza pandemic was caused by a novel lineage formed by reassortment between viruses from different hosts but that still contained HA and NA proteins of the H1 and N1 subtypes.^182^ Diverse names were used to refer to this new lineage, resulting in confusion.^183^ Complicating this issue was the fact that influenza A/(H1N1) viruses circulated in humans prior to 2009, but this novel lineage was genetically and antigenically distinct from those previously circulating viruses, despite belonging to the same subtype.^184^ To address this confusion and distinguish this lineage from influenza A/(H1N1) viruses that had been circulating before 2009, the nomenclature A/(H1N1)pdm09 was recommended.^185^

The categorical names assigned to groups of viruses within a subtype also evolve over time to account for antigenic drift within the subtype. Seasonal influenza A/(H1N1)pdm09 and A/(H3N2) viruses circulating since 2009 are divided into clades, which are typically defined by amino acid substitutions that differentiate progeny from ancestral viruses.^186^ The names of these clades and subclades are nested (eg clade 3C, having subclades 3C.1, 3C.2, and 3C.3, with 3C.2 having subclades 3C.2a and 3C.2b, and 3C.2a having the subclade 3C.2a1).^187^ Despite this subdivision into clades, though, viruses within them still belong to their respective subtypes, as their HA proteins more closely resemble those within the subtype compared to those of other subtypes (eg H3 compared to H1).

#### III.B.3. Knowledge at the Filing Date Governs Claim Construction

The meaning of claim terms is grounded in the art’s understanding at the time of filing, as illustrated by *Schering*.^188^ The patent at issue was originally filed as an application referring to ‘leukocyte interferon’.^189^ Six months after filing, however, new terminology was adopted because the term ‘leukocyte interferon’ reflected the source of the protein but did not distinguish the protein itself from other interferons, and leukocytes produce multiple types of interferon.^190^ The application at issue was then amended to substitute ‘IFN-α’ for ‘leukocyte interferon’.^191^^,^^192^

It was only later that scientists discovered interferon had multiple subtypes and that the originally named ‘IFN-α’ had subtypes including IFN-α1, IFN-β2, etc.^193^ Because the existence, let alone identity, of polypeptides of these other interferon subtypes was unknown, the Federal Circuit concluded that those polypeptides of other subtypes could not be within the scope of the ‘901 patent’s claims.^194^ The court therefore affirmed the district court’s conclusion that, because the specification did not differentiate between different subtypes of interferon, only one subspecies of IFN-α was described and enabled.^195^

The limited construction in *Schering* may be partly explained by the field’s nascent understanding of interferon at the relevant filing date, where scientists had no knowledge of the other polypeptides that could today be encompassed within ‘IFN-α’. Even alternatives known at the time of filing, however, but being understood as not falling within a term’s scope, will not be encompassed if the term later acquires a broader meaning.^196^ The *Kopykake* court, for example, considered whether the term ‘screen printing’ an edible image onto an edible foodstuff encompassed an accused infringer’s method of using ink jet printing for printing edible images.^197^ While ink jet printing existed at the time the patent application was filed and the patentee presented evidence that hundreds of thousands of ink jet printers had been sold,^198^ the district court determined that it was ‘an emerging technology just coming into its own as a method of printing on paper’, and it ‘strains credulity’ to suggest that it was a *conventional* means of applying images onto foodstuffs at the time of filing.^199^ The patentee also cited two patents related to ink jet printing of labels on pharmaceutical tablets or citrus fruits, but the Federal Circuit determined that these references did not establish that ink jet printing was a *conventional* method of printing images on foodstuffs at the filing date and affirmed the district court’s construction.^200^

While the *Kopykake* patentee declined to pursue infringement based on the doctrine of equivalents,^201^ such a result may have turned on whether ink jet printing, known at the time of filing, had been described in the patent. Alternatives that are disclosed, but not claimed, cannot be recaptured under the doctrine of equivalents.^202^ In *Johnson Johnston*, the *en banc* Federal Circuit reiterated its holding in *Maxwell* that when a patentee discloses but declines to claim subject matter, that unclaimed subject matter is dedicated to the public.^203^ To hold otherwise would allow applicants to present broad disclosures with narrow claims, avoiding examination of a broader scope, and later expanding exclusivity beyond what was properly examined.^204^ Following this rationale, the court overruled its earlier *YBM Magnex* decision,^205^ which had purported to limit *Maxwell* to situations where an unclaimed alternative was distinct from the claimed invention, and held that the doctrine of equivalents could not be invoked to extend an *aluminum* limitation to cover a disclosed but unclaimed *steel* substrate.^206^

Absent a controlling definition, disclosed examples of a term otherwise undefined in the specification or art, such as biological deposits, may be representative of a term’s meaning, with claim construction accounting for the diversity among such examples. *Intervet* concerned a patent relating to a newly discovered type of porcine circovirus, dubbed ‘PCV-2’ for ‘porcine circovirus type II’, which was pathogenic, in contrast to previously known, non-pathogenic porcine circoviruses belonging to ‘type I’ or ‘PCV-1’.^207^ The patentee had deposited five representative strains as part of its description, which formed the basis for a district court construction limiting the ‘porcine circovirus type II’ to the five deposited sequences.^208^ The Federal Circuit, however, considered the 96% homology among these representative PCV-2 sequences—in contrast to 76% homology to the example PCV-1 genome—and the patent’s reference to strains of the invention having ‘significant serological similarity’ to those deposited sequences, to represent a category of porcine circovirus being defined by similar pathogenicity and 96% homology, reversing the district court’s construction.^209^ By the time this decision was made, over 10 years after the application was filed, analysis of 148 genomes had identified PCV-2 as separating into two distinct clades 1 and 2, with subclades 1A, 1B, 1C, 2A, 2B, 2C, 2D, and 2E.^210^ Analysis of a randomly selected genome from each subclade shows that genomes in clade 1 do not necessarily share 96% sequence identity with those in clade 2 but are close at 95.2–95.4%.^211^ The separation between these genomes and PCV-1 was still unambiguous, though.^212^ An alternative construction not tied to the commonalities of the deposited genomes may thus have been broad enough to encompass even these later-discovered PCV-2 genomes while still being distinct from the prior art.

#### III.B.4. Applicants May Provide a Controlling Method of Identifying an Organism as Claimed

To support continuing applicability of a fixed specification over a 20-year term, applicants may consider expressly describing the steps an artisan should take to identify an organism as belonging to the category as defined in the specification. Such a description would follow logically from a controlling definition as discussed in Section III.A. Whereas that section concerned defining terms based on what was knowable at the time of filing, the relevant description here is prospective, outlining how an organism that may not have been identified, or even exist yet, should be analyzed to determine whether it meets a claim term. For example, a phylogenetic definition may be followed by a description of relevant published sequences and tree construction parameters that would allow the skilled person to place a newly identified sequence in a phylogenetic tree and determine the clade to which it belongs. An antigenic definition, alternatively, may be followed by a description of a relevant method for vaccinating animals and evaluating their sera against different antigens, to determine whether an organism shares a given degree of antigenic similarity with one described in the specification. Even further clarity may be provided by an exemplary analysis for a set of ingroup examples and a closest outgroup such as the deposited PCV-2 sequences and PCV-1 counterexample in *Intervet*. Extending that example, forming a tree using a newly isolated virus’s genome in combination with the disclosed ingroup and closest outgroup examples should allow unambiguous identification of the new isolate as belonging to the ingroup of interest or an outgroup such as a sister clade.

Again, the recommendation to define key terms and provide a detailed description to support them may seem obvious. But, as before, the same temptation exists to avoid characterizing a claim term in too much detail and being held to unintentionally limiting statements, particularly where classifications may be considered well known and not the point of novelty. It would be folly to assume, however, that understanding of organismal biology, classification schemes, and even the names used for certain organisms will remain fixed over 20 years. If a specification is to be fixed in time while biology moves forward, then providing forward-looking description to inform future artisans may reduce uncertainty in evaluating later infringement, even if the original terminology becomes outdated by that point or if previously unknown organisms may fall within the scope of definitions used at the time of filing. As Section III.C illustrates, applicants need not describe or enable subject matter that does not exist at the time of filing, but showing that later-arising subject matter infringes may turn on whether claims broad enough to encompass it are adequately described and enabled at their earlier filing date.

### III.C. Implications of Nomenclature May Evolve over 20 Years of a Patent’s Term

#### III.C.1. The Number of Known Organisms within a Given Taxon Increases over Time, as More are Discovered or Produced by Evolution

The implications of a given bacterial name may change as sampling and characterization reveal more information about diversity within a taxon. The International Code of Nomenclature for Prokaryotes mandates that a taxon’s name has no status unless it is validly published.^213^ Requirements for a valid publication include a description of the taxon’s properties and a ‘type’, the element with which the taxon’s name is permanently associated.^214^ The type is not necessarily the most typical or representative element of the taxon, but at least provides a fixed example of a bacterium within the taxon.^215^ As new bacteria are isolated and assigned to a given taxon, the field’s understanding of diversity within the taxon will thus evolve. Wang *et al.*, for example, described a novel *C. butyricum* isolate, B10, including its phenotypic properties as compared to the type strain VPI 3266.^216^ This description was published in 2018, over a century after the earliest publication of the species *C. butyricum* in 1880,^217^ and so, it is possible that the diversity within a taxon may never be *fully* characterized.

Implications of a given name also change as more is learned about bacteriology and known organisms are further characterized. Notwithstanding the reclassifications discussed in Section III.B, the shift from phenotypic characterization, to molecular-based methods of DNA–DNA reassociation and 16S rRNA gene sequence similarity, to the availability of full genome sequencing, has provided progressively more information about named bacteria that may have been isolated decades ago, such as the *C. butyricum* type strain VPI 3266.^218^

Turning to influenza viruses, antigenic drift produces novel viruses over time as immune selection favors HA and NA glycoproteins that let viruses evade pre-existing immunity.^219^ Despite changes in the circulating virus populations over time, the names of subtypes to which these viruses are assigned generally remain the same, as influenza A/(H3N2) viruses have circulated since 1968.^220^ It is thus expected that future seasonal epidemics will be caused by viruses in the same subtypes^221^ but whose genome sequences and their encoded HA and NA protein sequences are not knowable in advance. To address this known unpredictability of influenza virus evolution, public health authorities make annual recommendations of candidate vaccine viruses for use in upcoming seasonal vaccines.^222^ Vaccine manufacturers may then substitute previous season’s viruses for the most recently recommended set.^223^

#### III.C.2. The State of the Art and Understanding at the Filing Date Govern Enablement and Written Description

The relevant state of the art for enablement is that existing at the date of filing a patent application.^224^ In *Hogan*, the Federal Circuit’s predecessor considered the implications of allowing a later-existing state of the art to affect enablement of an earlier-filed claim.^225^ The application of *Hogan* disclosed how to make crystalline polymers of 4-methyl-1-pentene, but the Board determined that a rejected claim ‘encompasse[d] an amorphous polymer, which is manifestly outside the scope of the enabling teaching present in the case’.^226^ The amorphous polymers on which this finding relied, however, did not exist until 1962, nine years after the application’s 1953 filing date.^227^ The court held that if sufficient enablement exists at the time of filing, then enablement is established for all time, which cannot be changed by a later change in the state of the art.^228^

While later-existing embodiments need not be enabled, alternatives that are known at the filing date must be, if they fall within a claim’s scope.^229^ Flowering plants, as one example, are broadly divided into two categories, based on whether a seedling produces one leaf (monocotyledons or monocots) or two (dicotyledons or dicots).^230^  *Plant Genetic Systems* concerned a patent relating to genetically modified plants, seeds, and cells, in which all working examples were dicots, such as potatoes, tomatoes, and tobacco.^231^ At issue were whether claims to plant cells were enabled for monocots, and whether claims to plants and seeds were interpreted to exclude monocots, because the accused infringing product was corn, a monocot.^232^ The Federal Circuit affirmed findings of non-enablement of the *plant cell* claims, noting that at the filing date, monocots were known and stably transformed monocot cells were highly desirable, but the patent gave no instructions on how to produce them.^233^ With respect to the *plant* and *seed* claims’ construction, the patentee had added the limitation ‘susceptible to infection and transformation by Agrobacterium and capable of regeneration’ during prosecution to address a rejection of non-enablement for monocots, including corn, in which the examiner cited lack of evidence of monocot transformation.^234^ The court concluded that from this prosecution history alone, the claims could not cover monocots and affirmed the construction as excluding monocots.^235^

Beyond enabling those skilled in the art to implement the invention, a specification must also contain a written description that allows those of ordinary skill to recognize that the inventor had actually invented what is claimed.^236^ As with enablement, satisfaction of the written description requirement is tied to the filing date.^237^  *In re Entresto*, for example, concerned claims directed to a pharmaceutical composition comprising the AT 1-antagonist valsartan and NEP inhibitor sacubitril ‘administered in combination’, where the drug Entresto and accused generic forms comprise a ‘complex’ of non-covalently bonded valsartan and sacubitril.^238^ There, the Federal Circuit rejected generic manufacturers’ written description challenge, noting that that complex, which was not discovered until four years after the patent’s priority date, is not what is claimed, reversing the district court’s finding that the claims lacked written description.^239^

As with enablement, even where one structure is defined in the specification, written description of the category *as claimed* is required.^240^  *AbbVie Deutschland* concerned written description of claims reciting fully human antibodies that bind and neutralize IL-12, where the patent had disclosed only antibodies with VH3 heavy chains and lambda light chains, each being derived from a single lead antibody Joe–9.^241^ The Federal Circuit agreed with the jury’s finding that this limited class of structurally similar antibodies did not represent the full variety or scope of the genus of fully human anti-IL-12 antibodies.^242^ In particular, all of the disclosed antibodies shared at least 90% sequence similarity in the variable regions, whereas the accused infringing antibody differed considerably, sharing less than 50% sequence similarity with the Joe–9 antibodies and had distinct heavy and light chains (VH5 v. VH3, and kappa v. lambda, respectively), but still fell within the scope of the broad functional claims.^243^ Moreover, the jury heard expert testimony that antibodies with as much as 80% sequence similarity to Joe–9 could still bind different antigens, illustrating unpredictability in the field.^244^

Finally, the written description requirement applies to claims as a whole, including features that do not form the basis for novelty and inventiveness.^245^ The patent at issue in *Juno v. Kite*, for example, concerned a chimeric antigen receptor including a novel combination of a CD3ζ signaling domain and CD28 costimulatory region comprising a specific amino acid sequence but also included the claim limitation of a single-chain variable fragment (scFv) that ‘specifically interacts with a selected target’.^246^ The Federal Circuit determined that the patent disclosed only two scFv examples, however, and provided no details of characteristics, sequences, or structures that would allow the skilled artisan to determine which scFvs would bind which target, and reversed a jury verdict of adequate written description as being unsupported by substantial evidence.^247^ The court likened this patent to that in *AbbVie Deutschland*, as not providing meaningful guidance about which scFv will bind which target.^248^

#### III.C.3. Applicants May Describe the Historical and Present Diversity within Relevant Taxa

To bolster written description and enablement for claims encompassing more general categories of organisms, such as a subtype, species, or genus, applicants may consider supporting definitions and examples by reference to authoritative descriptions of relevant taxa. A taxon is useful insofar as it circumscribes a group of organisms sharing a certain degree of commonality.^249^ To patentees, this circumscription allows for claims encompassing a group of organisms that may be expected to provide similar utility to an organism with which an invention was actually reduced to practice. Where an inventor has knowledge of the biology relevant to their invention, and taxa are defined by conservation of certain biological features, linking the biology underpinning the invention to the extent at which it is conserved may help convey that the inventor had identified the group of organisms where the invention could be practiced (supporting written description) and increase predictability in applying it across that group (supporting enablement).

Having identified relevant taxa and their associated descriptions in the literature, applicants may also consider enumerating examples of organisms representing the diversity existing within those taxa. A taxon with an existing definition and description is dense, in that organisms can be isolated from nature and assigned to the taxon based on their observable properties, increasing the number of known organisms within it over time.^250^ Even if an organism has not yet been identified, it would still *belong* to the taxon once so characterized,^251^ and so the true size of a taxon is both vast and potentially unknowable at a given time. The same may not be true of the *diversity* encompassed by the taxon so defined, since newly isolated organisms may be very similar to those already known, or at least within established boundaries.^252^ Description of examples representing a taxon’s diversity would further support claims encompassing general categories of organisms suitable for practicing an invention, even where future artisans seeking to implement the invention may consider isolating and using new organisms within those categories.

Examples of diversity within taxa should ideally account for the historical dimension of diversity. There is continued turnover in influenza virus populations, with newer clades replacing older ones, but the A/(H3N2) subtype classification still applies to a group of influenza A viruses that have been circulating since 1968.^253^ The diversity of viruses within the A/(H3N2) subtype is thus broader than any population of viruses circulating at any one time.^254^ Rather, the term encompasses a population that has been evolving for decades and will continue to evolve over the next 20 years.^255^ It is this continued evolution that leads public health authorities to update the influenza virus isolates recommended for inclusion in seasonal vaccines.^256^ Continued relevance of claims related to evolving populations, then, depends on their encompassing members of the population generally, including organisms that do not yet exist, rather than an extant organism in which an invention is reduced to practice. The following Section III.D considers the written description and enablement requirements in light of that scope.

### III.D. Claims Encompassing Categories of Organisms Require Written Description and Enablement across the Category’s Full Scope

#### III.D.1. The Extent of Commonality between Organisms in a Group Will Vary across Taxonomic Scales and the Underlying Classification Scheme

In general, the more closely related two organisms are, the more similar they are genotypically and phenotypically. In bacteriology, Bergey’s Manual of Determinative Bacteriology classified bacteria in a nested hierarchy from phylum to species, each rank representing increasing relatedness and phenotypic similarity.^257^ More recently, the Genome Taxonomy Database organizes taxonomic ranks by relative evolutionary divergence between genome sequences, which increases with progression from species to phylum.^258^ The hierarchical taxonomy recently ratified by the International Committee on Taxonomy of Viruses similarly orders viruses in a hierarchy from realm to species, with intra-taxon divergence decreasing with progression from realm to species.^259^ The genus *Betacoronavirus*, for example, encompasses SARS-CoV, MERS-CoV, and SARS-CoV-2, but each belongs to a separate species under this hierarchical taxonomy, with varying accessory genes observed between different members of the genus.^260^ In such hierarchical taxonomies, evolutionary divergence decreases, and genetic similarity increases, with progression from higher taxa (phylum or realm) to lower taxa (species).

In bacteria, multiple independent investigations have observed increased genetic and phenotypic similarity as relatedness increases. Bauer *et al.*, for example, observed increasing metabolic similarity between bacteria of progressively lower taxa, with the highest similarity being within a species.^261^ As one example of such metabolic similarity, Martiny *et al.* describe enrichment of *Bacteroides* species in the guts of individuals with protein- and fat-rich diets, whereas *Prevotella* species are enriched in those with carbohydrate-rich diets.^262^ The genera *Prevotella* and *Bacteroides* both belong to the phylum Bacteroidota (formerly Bacteroidetes),^263^ and so, less similarity may be found running throughout higher taxonomic ranks. Similarly, Martiny *et al.* note that bacterial glycoside hydrolase sequences are conserved at the genus and species levels, but family is poorly predictive of the abundance of such enzymes in bacterial genomes.^264^

Despite not defining a taxon *per se*, 16S rRNA gene sequence similarity also provides some indication of phylogenetic and phenotypic similarity. Building on large data sets characterizing the presence or absence of different traits among multiple bacteria, Martiny *et al.* derived a metric of trait depth (τD), the average 16S rRNA sequence distance between (i) members of a clade, where at least 90% of strains in the clade exhibit a given trait and (ii) the root node of this clade. ^265^ Sevigny *et al.* subsequently analyzed the relationship between 16S rRNA gene sequences and shared protein-coding gene content and observed that bacterial genomes sharing 16S rRNA gene sequences with at least 99% identity shared on average 78% of protein-coding genes.^266^ While the authors qualified this result by noting that 22% of functional capacity cannot be accounted for by 16S rRNA gene sequence alone, the 78% that *is* accounted for may still be meaningful depending on an invention’s context, if an absolute or invariable correlation is not required for patentability.^267^

Turning to viruses, the applicability of mutations from one virus background depends on conservation of the features underlying the utility of mutations. Influenza virus HA proteins, for example, contain pH-sensitive histidine residues that have been proposed to act as molecular switches, triggering fusion of the viral envelope with the endosomal membrane at low pH to allow release of viral genome segments into the cytoplasm.^268^ While certain conserved histidine residues have been suggested as potential switches, some are not universally conserved among subtypes, and studies investigating those residues have yielded contradictory results.^269^ On the other hand, certain mutations have been identified to stabilize HA proteins of multiple subtypes in a prefusion conformation—Byrd-Leotis *et al.* described a K58I substitution that conferred acid stability for nearly all tested IAV HAs,^270^ while Milder *et al.* of Janssen Vaccines reported stabilization of all tested IAV HAs by substitutions in two pH-sensitive regions, H26W in the first and K51I + E103I in the second.^271^ The Janssen Vaccines group later reported a distinct approach to stabilize pH sensitivity of influenza B virus HA proteins, noting the limited sequence similarity and substantial structural differences between IAV and IBV HA proteins underlying the two different approaches.^272^

#### III.D.2. Written Description and Enablement Require Support across the Scope as Claimed, in View of Background Knowledge in the Art

As Karshtedt *et al.* review in *The Death of the Genus Claim*, generalization of one or a few examples to a functional or overly broad category poses challenges for written description.^273^ In *Eli Lilly*, for example, a nucleotide sequence of rat insulin-encoding complementary DNA (cDNA) did not constitute an adequate description of mammalian insulin cDNA generally, or even human insulin cDNA narrowly.^274^ In *Ariad*, the Federal Circuit similarly held invalid claims encompassing use of all substances that achieve the desired result of inhibiting binding of NF-κB to its recognition sites.^275^ Lastly, the *Juno* court reversed a jury verdict of adequate written description for the genus of ‘a binding element that specifically interacts with a selected target’, where only two examples were disclosed.^276^

Despite the challenges posed by *Eli Lilly*, *Ariad*, and *Juno*, genus claims may still be supported by classifications tied to disclosed examples and background knowledge in the art. Both *Curtis* and *Rochester*, affirming lack of written description, distinguished analogous cases, *Smythe* and *Unocal* respectively, where written description was satisfied based on how those skilled in the art would have understood the disclosed examples to extend to the claimed genera. In *Curtis*, the Federal Circuit affirmed the lack of written description for a genus of ‘friction-enhancing coatings’ where the specification attributed unique properties to the disclosed species of microcrystalline wax, such that no other coatings would naturally occur to those of ordinary skill.^277^ The *Curtis* court distinguished *Smythe*, where disclosure of ‘an inert gas’ as a segmentizing medium to separate liquid samples supported a claimed genus of ‘an inert fluid’, which could include an inert liquid, because the specification clearly conveyed that the characteristics of a fluid are what make the segmentizing medium work.^278^ And in *Rochester*, the Federal Circuit affirmed the district court’s finding that claims reciting ‘a compound that selectively inhibits PGHS-2 activity’ lacked adequate written description because the specification did not disclose any compounds that could be used in the claimed methods, and there was no evidence that any such compounds were known.^279^ The *Rochester* court distinguished *Unocal*, in which written description was satisfied for claims that ‘specif[ied] the chemical properties of the gasolines, reflecting the way oil refiners formulate gasoline’.^280^ Even if the claims specified desired properties, those skilled in petroleum refining knew how to mix streams of raw petroleum sources to achieve such desired properties.^281^

The results in *Unocal* and *Smythe* underscore the importance of the specification being read from the background knowledge of one skilled in the art, as illustrated by two more recent life science cases. In *Ajinomoto*, the Federal Circuit affirmed a finding of adequate written description for the genus of ‘more potent promoter[s]’, in view of a well-known link between consensus sequence and promoter strength, and the genus having been well explored before the priority date.^282^ And shortly after *Juno*, the court decided *BASF*, which concerned written description of claims concerning plant cells that produce omega-3 long-chain polyunsaturated fatty acids (LCPUFAs).^283^ The patent at issue described successful LCPUFA production in *Arabidopsis*, a model organism, but at issue was whether possession was satisfied for LCPUFA production in another species, *B. napus* (rapeseed, a cultivar of which is used for canola oil production), and for plants generally.^284^ The court determined that substantial evidence supported the jury’s finding that skilled artisans would have expected the invention to work in *B. napus*, given expert testimony that *Arabidopsis* and *B. napus* shared the ability to produce ALA, the starting point for the Δ6-desaturase pathway, so that ‘if you had [the invention] in *Arabidopsis*, then you had it in canola’.^285^ The parties had ‘not given [the court] much to work with’ regarding the broader genus claims directed to any plant cell, though, with BASF conceding that it had made almost no invalidity arguments against those claims if it was not successful against the narrower canola claims.^286^ Still, the Federal Circuit seemed to go out of its way to overturn the jury verdict of adequate written description for those claims, on the basis that CSIRO’s rebuttal evidence supporting the leap from *Arabidopsis* to *B. napus* did not extend to all plant cells.^287^

In addition to a written description, the specification must enable those of skill in the art to make and use the claimed invention.^288^ In *Wands*, the Federal Circuit set forth factors to assess whether any experimentation needed to practice an invention is reasonable, or undue experimentation weighing against enablement.^289^ These factors include at least the breadth of the claims, state of the prior art, level of predictability in the art, amount of direction provided by the inventor, and quantity of experimentation needed.^290^

As Lemley & Sherkow review in *The Antibody Patent Paradox*, enablement poses particular challenges for antibody claims that are broader than disclosed antibody structures or complementarity-determining regions (CDRs), such as those claiming a genus of antibody that binds to a particular target and has a certain function.^291^  *Amgen*, for example, concerned a genus of antibodies that was not limited to any of the 26 antibodies the patentee had produced, or even their CDRs, but rather an antibody that binds at least two of a list of specified residues of PCSK9 ‘and blocks the binding of PCSK9 to LDLR by at least 80%’.^292^ Amgen had proffered methods of making such antibodies through conservative substitutions to its disclosed antibodies, or *de novo* immunization and screening, but the Supreme Court considered these approaches to ‘amount to little more than two research assignments’ and held that Amgen’s disclosure failed to enable all that it had claimed.^293^

While Amgen’s functional genus claims were not enabled, the Court did note that enablement is not limited to working examples, and it may suffice to provide ‘some general quality running through’ a claimed category that gives it ‘peculiar fitness for the particular purpose’.^294^ Such a common structural feature was lacking in *Baxalta*, where the Federal Circuit applied *Amgen* to affirm a summary judgment of lack of enablement of claims directed to a genus of antibody ‘that binds Factor IX or Factor IXa and increases the procoagulant activity of Factor IXa’.^295^

Intermediate taxa, characterized by less diversity and more shared features, may be more likely to share such common qualities, and offer stronger enablement than higher, more diverse taxa or wholly functional categories. In *Vaeck*, for example, the Federal Circuit affirmed an enablement rejection of claims encompassing heterologous gene expression in the large, diverse, and poorly understood group of Cyanobacteria generally, but reversed the same rejection of a dependent claim limited to the genera *Anacystis* and *Synechocystis*.^296^ And, in *Martek Biosciences*, concerning fermentation of euryhaline microorganisms, a district court granted judgment as a matter of law that all claims were invalid as lacking enablement.^297^ While ‘euryhaline microorganisms’ as recited in an independent claim encompassed potentially 10,000 organisms, and the record reflected ‘a lot of unpredictability’ and ‘an enormous amount of research’ to find a euryhaline organism that would satisfy claim 1, dependent claims were limited to the order Thraustochytriales, or to the genera *Thraustochytrium* and *Schizochytrium*, which together encompass only 22 species.^298^ The court reasoned that the expert testimony regarding the quantity of experimentation is far less relevant and persuasive with respect to these narrower groups and so reversed the district court’s judgment of non-enablement for these dependent claims.^299^

#### III.D.3. Applicants May Link a Principle Underlying the Invention to the Depth at Which Organismal Features Relevant to that Principle are Conserved

Despite the modern nature of antibody technology, the *Amgen* decision was grounded in the longstanding, fundamental *quid pro quo* of the patent bargain—one must enable what one has claimed.^300^ Similarly, the above written description cases consistently note that the specification must allow those skilled in the art to recognize that the inventors *had actually invented* what is now claimed.^301^ As those cases illustrate, though, that is not to say that patentees are limited to narrowly claiming what they have actually reduced to practice.

To support generalization of a working example’s organism to claim scope consistent with knowledge in the art, applicants may consider describing a principle underlying the operation of an example actually reduced to practice, the organismal properties involved in that principle, and the depth at which those properties are conserved. The result in *BASF* may be contrasted with *Eli Lilly*, for example, by the conservation of the relevant pathways between *Arabidopsis thaliana*, the model plant actually reduced to practice, and *B. napus*, the commercially valuable species.^302^ While some patents at issue in *BASF* had claims reciting the intermediate taxon *Brassica* between the species *B. napus* and the kingdom of plant,^303^ the court’s opinion distinguished only the ‘canola claims’ reciting *B. napus* from the ‘broader genus’ claims reciting ‘plant’ generally.^304^ The credit afforded to conservation of the relevant pathways between *A. thaliana* and *B. napus* raises the questions, though, of how conserved such pathways are in the genus *Brassica* or family Brassicaceae containing both *A. thaliana* and *Brassica*,^305^ and to what extent a generic claim to an intermediate taxon would have satisfied written description. As the genus *Brassica* contains other food crops, such as *B. oleracea* (broccoli, kale, cauliflower) and *B. rapa* (turnips),^306^ claims to such intermediate taxa may provide additional value where claims to much larger and diverse taxa are less likely to have adequate written description.

Description of such underlying principles and their depths of conservation may also provide evidentiary support for enablement of claims to related organisms that are expected to share the relevant properties. Without such a principle underlying an invention’s operation with one organism, it may be challenging to identify the properties of the organism involved in that operation, and consequently difficult to establish an expectation of success using other organisms. The Federal Circuit’s reversal of an enablement rejection of a dependent claim reciting two genera in *Vaeck* appears related to prior art knowledge of one species within one genus and a working example in one strain within one species of the other genus.^307^ The *Vaeck* court did not address, however, the diversity and predictability within the claimed genera relative to the species that provided evidence of enablement.^308^ And in *Martek Biosciences*, the court highlighted the limited size of the dependent claims’ genera relative to the large and unpredictable category of ‘euryhaline microorganism’ in reversing the non-enablement judgment of those dependent claims but did not address the relative predictability within those smaller genera.^309^ While the dependent claims in both cases may have offered smaller, less diverse genera than the broad and more unpredictable categories of the non-enabled independent claims, it is uncertain whether the diversity within even those narrower genera could sustain a more focused enablement challenge, absent some indication of conserved properties relevant to the inventions claimed.

## IV. CONCLUSION

The stringency of the written description and enablement doctrines in biotechnology appears, at the moment, here to stay. But so too does the relevance of organismal biology to inventions that patentees in life sciences seek to protect. If organisms, like antibodies, cannot easily be defined in functional terms of what they *do* while complying with these requirements, this underscores the importance of defining what they *are*. A tangible organism accessible following a biological deposit is one manner of definition, albeit a narrow one in a patent system that allows protection beyond physical embodiments actually reduced to practice. Systematics, being concerned with classifying, naming, and identifying organisms according to their evolutionary history and relatedness, is well suited to the task of defining and describing a group of organisms that biologists would recognize as closely related and phenotypically similar to those exemplified, bolstering written description and enablement of genus claims. And almost paradoxically, describing their evolutionary *history* that is inferred, if not observed, may mitigate the effects of *future* evolution that is unpredictable, but observable, on claim construction and infringement analyses. The very fields of evolutionary biology concerned with classifying organisms to account for their evolutionary history therefore offer an appropriate solution to the problems that evolution poses for patentees in the life sciences.

## Footnotes

1
*See* U.S. Centers for Disease Control and Prevention, *History of Measles*, Measles (Rubeola), https://www.cdc.gov/measles/about/history.html (May 9, 2024).

2
*See generally* Florian Krammer *et al.*, *Influenza*, 4 Nat.  Rev.  Dis. Primers 3 (2018) [hereinafter Krammer *et al.*].

3
*See generally* Yang Tan *et al.*, *Engineered Live Biotherapeutics: Progress and Challenges*, 15 Biotechnol. J. e2000155 (2020) [hereinafter Tan *et al.*]; *see generally* Jack A. Gilbert *et al.*, *Clinical translation of microbiome research*, 31 Nat. Med. 1099 (2025) [hereinafter Gilbert *et al.*]; *see generally* Han Fang *et al.*, *Postbiotic Impact on Host Metabolism and Immunity Provides Therapeutic Potential in Metabolic Disease*, 46 Endocr.  Rev. 60 (2025) [hereinafter Fang *et al.*].

4
*See generally* In re Vaeck, 947 F.2d 488 (Fed. Cir. 1991); *see generally* BASF Plant Sci., LP v. Commonwealth Sci. & Indus. Rsch. Organisation, 28 F.4th 1247 (Fed. Cir. 2022); *see generally* Diane Wray-Cahen *et al.*, *Advancing Genome Editing to Improve the Sustainability and Resiliency of Animal Agriculture*, 3 CABI Agric.  Biosci. 21 (2022).

5
*See generally* Vaeck; *see generally* BASF.

6
*See* 35 U.S.C. § 112(b).

7
*See* In re Papesch, 135 F.2d 381, 391 (C.C.P.A. 1963).

8
*Id.*

9 See generally Krammer *et al.*

10
*See generally* Joseph D. Dukes *et al.*, *The MDCK Variety Pack: Choosing the Right Strain*, 12 BMC Cell.  Biol. 43 (2011) [hereinafter Dukes *et al.*].

11
*See generally* Magdalena Malm *et al.*, *Evolution from Adherent to Suspension: Systems Biology of HEK293 Cell Line Development*, 10 Sci  Rep 18,996 (2020) [hereinafter Malm *et al.*]; *see also* F. L. Graham *et al.*, *Characteristics of a Human Cell Line Transformed by DNA from Human Adenovirus Type 5*, 36 J. Gen. Virol. 59 (1977).

12
*See generally* Dukes *et al.*; *see generally* Malm *et al.*

13
*See* In re Wands, 858 F.2d 731, 735 (Fed. Cir. 1988) (‘One means that has been developed for complying with the enablement requirement is to deposit the living materials in cell depositories which will distribute samples to the public who wish to practice the invention after the patent issues’.).

14
*See generally* Ian MacLeod, *The Scope and Infringement of Patents Claiming Strains of Bacteria*, 4 Access  Microbiol. acmi000449 (2022).

15
*Id.* at Abstract.

16
*See* Genzyme Corp. v. Transkaryotic Therapies, Inc., 346 F.3d 1094, 1097–1104 (Fed. Cir. 2003) (discussing the implications of depositing a recombinant vector on construction of the term ‘chromosomally integrated’); *but see id.* at 1109 (Linn, J., concurring in part and dissenting in part) (criticizing the majority’s view that the deposited vector remained necessary to the claims despite later removal of the term ‘recombinant vector’).

17
*See* Intervet Inc. v. Merial Ltd, 617 F.3d 1282, 1285–1287 (Fed. Cir. 2010) (discussing claim construction in relation to sequence diversity of deposited PCV-2 genomes).

18
*See generally* Fin G. Milder *et al.*, *Universal Stabilization of the Influenza Hemagglutinin by Structure-Based Redesign of the pH Switch Regions*, 119 Proc.  Natl.  Acad. Sci. U S A e2115379119 (2022) [hereinafter Milder *et al.*].

19
*See generally* Krammer *et al.*

20
*See* Magali Cordaillat-Simmons *et al.*, *Live Biotherapeutic Products: The Importance of a Defined Regulatory Framework*, 52 Exp.  Mol.  Med. 1397, 1401–1402 (2020).

21
*See generally* Ramon Rosselló-Mora & Rudolf Amann, *The Species Concept for Prokaryotes*, 25 FEMS Microbiol. Rev. 39 (2001) [hereinafter Rosselló-Mora & Amann]; *see generally* M. H. V. van Regenmortel, *Virus Species and Virus Identification: Past and Current Controversies*, 7 Infect.  Genet.  Evol. 133 (2007) [hereinafter van Regenmortel, *Virus Species and Virus Identification*].

22
*See generally* Donovan H. Parks *et al.*, *A Standardized Bacterial Taxonomy Based on Genome Phylogeny Substantially Revises the Tree of Life*, 36 Nat. Biotechnol. 996 (2018) [hereinafter Parks *et al.*, *A Standardized Bacterial Taxonomy*].

23
*See generally* World Health Organization, *Standardization of Terminology of the Pandemic A(H1N1)2009 Virus*, 86 Wkly. Epidemiol. Rec. 480 (2011) [hereinafter WHO].

24
*See generally* Schering Corp. v. Amgen Inc., 222 F.3d 1347 (Fed. Cir. 2000); *see generally* Mark A. Lemley, *The Changing Meaning of Patent Claim Terms*, 104 Mich. L. Rev. 101 (2005).

25
*See generally* Dmitry Karshtedt *et al.*, *The Death of the Genus Claim*, 35 Harv. J. L. & Tech. 1 (Fall 2021) [hereinafter Karshtedt *et al.*]; *see generally* Mark A. Lemley & Jacob S. Sherkow, *The Antibody Patent Paradox*, 132 Yale  L.  J. 994 (2023) [hereinafter Lemley & Sherkow].

26
*See generally* Karshtedt *et al.*

27
*See generally* Lemley & Sherkow; *see also id.* at 1055 (‘it’s not clear that sequence-homology claims would avoid condemnation in the Federal Circuit’s war on genus claims’.); *see also id.* at 1038–1040.

28
*See*, eg Michael T. Madigan *et al.*, *Brock Biology of Microorganisms* 377–390 (12th ed. 2008) [hereinafter Madigan].

29
*See* Jason Mast, *Want to Start a Fight among Virus Experts? Ask about HIV’s New Name*, STAT, https://www.statnews.com/2025/01/09/virology-new-scientific-names-for-hiv-covid-19-in-updated-viral-taxonomy (Jan. 9, 2025) (‘“Somebody needs to take away whatever drugs the committee is on,” wrote Kristian Andersen, a virologist at Scripps Institute, on Bluesky….Angela Rasmussen, an emerging pathogens researcher at the University of Saskatchewan, seemed ready to lay siege to Kuhn’s house. “Down with the ever-tightening iron-fisted grip of taxonomic dictators,” she wrote’.).

30
*See generally* Daniel M. Weinberger *et al.*, *Serotype Replacement in Disease after Pneumococcal Vaccination*, 379 Lancet 1962 (2011).

31 S*ee generally* Sarah E. Kidd *et al.*, *Fungal Nomenclature: Managing Change is the Name of the Game*, 10 Open  Forum  Infect. Dis. ofac559 (2023).

32
*See generally* Ralph E. Harbach, *The Culicidae (Diptera): A Review of Taxonomy, Classification and Phylogeny*, 1668 Zootaxa 591 (2007).

33
*See generally* Dmitry A. German *et al.*, *An Updated Classification of the Brassicaceae (Cruciferae)*, 220 PhytoKeys 127 (2023).

34
*See generally* Lindsay S. Miles *et al.*, *Urban Evolution Comes into Its Own: Emerging Themes and Future Directions of a Burgeoning Field*, 14 Evol. Appl. 3 (2020); *see generally* Ken A. Thompson *et al.*, *Speciation and the City*, 33 Trends  Ecol. Evol. 815 (2018).

35
*See generally* Peter H. Thrall *et al.*, *Evolutionary Change in Agriculture: The Past, Present and Future*, 3 Evol. Appl. 405 (2010); *see generally* Martin M. Turcotte, *The Eco-evolutionary Impacts of Domestication and Agricultural Practices on Wild Species*, 372 Philos. Trans. R. Soc. Lond. B Biol. Sci. 20160033 (2017).

36
*See generally* Nathan P. Wiederhold, *Emerging Fungal Infections: New Species, New Names, and Antifungal Resistance*, 68 Clin. Chem. 83 (2021).

37
*See generally* Rosselló-Mora & Amann (discussing bacteria); *see generally* van Regenmortel, *Virus Species and Virus Identification* (discussing viruses).

38
*See generally* BASF (concerning canola, a cultivar of rapeseed).

39
*See generally* Atsushi Yoshiki and Kazuo Moriwaki, *Mouse Phenome Research: Implications of Genetic Background*, 47 ILAR J. 94 (2006); *see generally* Roel Meyermans *et al.*, *Genetic and Genomic Analysis of Belgian Blue’s Susceptibility for Psoroptic Mange*, 56 Genet. Sel. Evol. 52 (2024).

40
*See generally* Dukes *et al.*; *see generally* Malm *et al.*

41
*See generally* Pablo Engel *et al.*, *CD Nomenclature 2015: Human Leukocyte Differentiation Antigen Workshops as a Driving Force in Immunology*, 195 J. Immunol. 4555 (2015).

42 Charles A. Janeway, Jr *et al.*, *Immunobiology: The Immune System in Health and Disease* (5th ed. 2001), Appendix II. CD Antigens.

43
*See generally* Mathilde Foglierini *et al.*, *AncesTree: An Interactive Immunoglobulin Lineage Tree Visualizer*, 16 PLoS. Comput. Biol. e1007731 (2020).

44
*See generally* Sarah Christensen *et al.*, *Detecting evolutionary patterns of cancers using consensus trees*, 36 Bioinformatics i684 (2020).

45 Madigan at 385.

46
*Id.*; W. Ludwig & K. H. Schleifer, *Bacterial Phylogeny Based on 16S and 23S rRNA Sequence Analysis*, 15 FEMS Microbiol. Rev. 155, 155–156 (1994) [hereinafter Ludwig & Schleifer].

47 Madigan at 394; Ludwig & Schleifer at 155–156.

48 Madigan at 394; Ludwig & Schleifer at 155–156.

49 Ludwig & Schleifer at 155–156.

50 Madigan at 385 (citing Charles A. Darwin, *On the Origin of Species* (1st ed. 1859) (‘Our classifications will come to be, as far as they can be so made, genealogies’)).

51
*See* Madigan at 377–381; *see* Roderic D. M. Page & Edward C. Holmes, *Molecular Evolution: A Phylogenetic Approach* 1–11 (1998) [hereinafter Page & Holmes].

52 Madigan at 371, 380–381; Page & Holmes at 11.

53 Madigan at 381; Page & Holmes at 28.

54
*See* Page & Holmes at 29.

55
*See id.*

56
*See id.*

57
*See generally* Jian-Yu Jiao *et al.*, *Advancements in Prokaryotic Systematics and the Role of Bergey's International Society for Microbial Systematics in Addressing Challenges in the Meta-Data Era*, 11 Natl. Sci. Rev. nwae168 (2024) [hereinafter Jiao *et al.*] (reviewing the history of prokaryotic systematics).

58
*See id.* at 2 (‘Widely acknowledged as the “Bible” of prokaryotic taxonomy, [Bergey’s Manual] aims to offer detailed descriptions of the systematics, ecology, physiology, morphology and other biological properties of all described prokaryotes’.).

59 Noel R. Krieg *et al.*, *Bergey’s Manual of Systematic Bacteriology: Volume 2: The Proteobacteria, Part B: The Gammaproteobacteria*, 607 (2nd ed. 2010).

60
*See* Jiao *et al.* at 3.

61
*See generally* L. G. Wayne *et al.*, *Report of the Ad Hoc Committee on Reconciliation of Approaches to Bacterial Systematics*, 37 Int. J. Syst. Bacteriol. 463 (1987) [hereinafter Wayne *et al.*].

62
*See generally* Carl R. Woese & George E. Fox, *Phylogenetic Structure of the Prokaryotic Domain: The Primary Kingdoms*, 74 Proc. Natl. Acad. Sci. U S A 5088 (1977) [hereinafter Woese & Fox].

63
*See generally* E. Stackebrandt & B. M. Goebel, *Taxonomic Note: A Place for DNA–DNA Reassociation and 16S rRNA Sequence Analysis in the Present Species Definition in Bacteriology*, 44 Int. J. Syst. Bacteriol. 846 (1994) [hereinafter Stackebrandt & Goebel].

64
*Id.* at 849.

65
*See generally* Erko Stackebrandt & Jonas Ebers, *Taxonomic Parameters Revisited Tarnished Gold Standards*, 33 Microbiology  Today 152 (2006) [hereinafter Stackebrandt & Ebers].

66
*See* Jethro Johnson, *Finding a New Role for 16S rRNA Gene Sequencing in Microbiome Research, Springer Nature Research Communities*, Springer Nature Research Communities, https://communities.springernature.com/posts/finding-a-new-role-for-16s-rrna-gene-sequencing-in-microbiome-research (Nov. 12, 2019).

67
*See id.*

68
*See* Jiao *et al.* at 3.

69
*See generally* Konstantinos T. Konstantinidis & James M. Tiedje, *Genomic Insights That Advance the Species Definition for Prokaryotes*, 102 Proc. Natl. Acad. Sci. U S A 2567 (2005).

70
*See generally id.*

71
*See generally id.*

72
*See generally* Johan Goris *et al.*, *DNA–DNA Hybridization Values and Their Relationship to Whole-Genome Sequence Similarities*, 57 Int. J. Syst. Evol. Microbiol. 81 (2007) [hereinafter Goris *et al.*].

73
*See generally* Chirag Jain *et al.*, *High Throughput ANI Analysis of 90 K Prokaryotic Genomes Reveals Clear Species Boundaries*, 9 Nat. Commun. 5114 (2018).

74
*See generally* Connor S. Murray *et al.*, *Re-evaluating the Evidence for a Universal Genetic Boundary among Microbial Species*, 12 Nat. Commun. 4059 (2021).

75
*See generally* Luis M. Rodriguez-R *et al.*, *Reply to: Re-evaluating the Evidence for a Universal Genetic Boundary among Microbial Species’*, 12 Nat. Commun. 4060 (2021).

76 Donovan H. Parks *et al.*, *A Complete Domain-to-Species Taxonomy for Bacteria and Archaea*, 38 Nat. Biotechnol. 1079 (2020) [hereinafter Parks *et al.*, *A Complete Domain-to-Species Taxonomy*].

77
*See generally id.*

78
*See generally id.*

79 Diamond v. Chakrabarty, 447 U.S. 303 (1980).

80 US Patent No. 4,259,444, col. 3 l. 63 – col. 4 l. 12 (citing R.Y. Stanier *et al.*, *The Aerobic Pseudomonads a Taxonomic Study*, 43 J. Gen. Microbiol. 159 (1966) [hereinafter Stanier *et al.*]).

81
*See* US Patent No. 8,476,056 (claiming priority to 2007).

82
*See* US Patent No. 5,629,468 (claiming priority to 1994).

83
*See* US Patent No. 6,187,569 (claiming priority to 1980).

84
*See* US Patent No. 11,969,446 (claiming priority to 2019).

85
*See* US Patent Application Serial No. 18/004,390 (claiming priority to 2020).

86
*See generally* M. H. V. van Regenmortel *et al.*, *Guidelines to the Demarcation of Virus Species*, 142 Arch. Virol. 1505 (1997); *see generally* Marc H. V. van Regenmortel & Brian W. J. Mahy, *Emerging Issues in Virus Taxonomy*, 10 Emerg. Infect. Dis. 8 (2004) [hereinafter van Regenmortel & Mahy]; *see generally* van Regenmortel; *see generally* Marc H. V. van Regenmortel, *The Species Problem in Virology*, 100 Adv. Virus  Res. 1 (2018).

87
*See generally* Michelle Wille & Edward C. Holmes, *The Ecology and Evolution of Influenza Viruses*, 10 Cold  Spring  Harb. Perspect. Med. a038489 (2020) [hereinafter Wille & Holmes].

88
*See generally id.*

89
*See generally id.*

90
*See generally id.*

91
*See generally id.*

92
*See generally id.*

93
*See generally id.*

94
*See generally* Nicole M. Bouvier & Peter Palese, *The Biology of Influenza Viruses*, 26 Suppl 4 Vaccine D49 (2008) [hereinafter Bouvier & Palese].

95
*See generally id.*

96
*See generally id.*

97
*See generally* Derek J. Smith *et al.*, *Mapping the Antigenic and Genetic Evolution of Influenza Virus*, 305 Science 371 (2004).

98
*See generally* Victor C. Huber *et al.*, *A multi-valent Vaccine Approach That Elicits Broad Immunity within an Influenza Subtype*, 27 Vaccine 1192 (2009) [hereinafter Huber *et al.*].

99
*See generally* James D. Allen & Ted M. Ross, *H3N2 Influenza Viruses in Humans: Viral Mechanisms, Evolution, and Evaluation*, 14 Hum. Vaccin. Immunother. 1840 (2018) [hereinafter Allen & Ross].

100
*See* Patricia A. Jorquera *et al.*, *Insights into the Antigenic Advancement of Influenza A(H3N2) Viruses, 2011-2018*, 9 Sci  Rep 2676 (2019) [hereinafter Jorquera *et al.*], Fig. 1.

101 Rosselló-Mora & Amann at 51.

102 The Linnean Society, *His Career and Legacy*, The Linnean Society of London, https://www.linnean.org/learning/who-was-linnaeus/career-and-legacy (accessed Apr. 12, 2025).

103
*See generally* Rosselló-Mora & Amann (discussing bacteria); *see generally* van Regenmortel, *Virus Species and Virus Identification* (discussing viruses).

104 Rosselló-Mora & Amann at 51.

105
*Id.* at 56.

106
*See id.*

107
*See generally* William P. Hanage *et al.*, *Fuzzy Species among Recombinogenic Bacteria*, 3 BMC Biol. 6 (2005).

108
*See* van Regenmortel, *Virus Species and Virus Identification* at 135–136.

109
*See generally* Anice C. Lowen, *It's in the Mix: Reassortment of Segmented Viral Genomes*, 14 PLoS Pathog. e1007200 (2018).

110
*See generally* Lin Chao, *Evolution of Sex in RNA Viruses*, 7 Trends  Ecol. Evol. 147 (1992).

111
*See generally* Nicolle Marshall *et al.*, *Influenza Virus Reassortment Occurs with High Frequency in the Absence of Segment Mismatch*, 9 PLoS Pathog. e1003421 (2013); *see generally* Nathan T. Jacobs *et al.*, *Incomplete Influenza A Virus Genomes Occur Frequently but Are Readily Complemented during Localized Viral Spread*, 10 Nat. Commun. 3526 (2019).

112
*See generally* Maria C. White *et al.*, *Heterologous Packaging Signals on Segment 4, but Not Segment 6 or Segment 8, Limit Influenza A Virus Reassortment*, 91 J. Virol. e00195 (2017); *see generally* Maria C. White *et al.*, *H5N8 and H7N9 Packaging Signals Constrain HA Reassortment with a Seasonal H3N2 Influenza A Virus*, 116 Proc. Natl. Acad. Sci. U S A 4611 (2019).

113
*See generally* Tongyu Liu *et al.*, *The Evolutionary Potential of Influenza A Virus Hemagglutinin Is Highly Constrained by Epistatic Interactions With Neuraminidase*, 30 Cell  Host  Microbe 1363 (2022).

114 Erko Stackebrandt *et al.*, *Report of the Ad Hoc Committee for the Re-evaluation of the Species Definition in Bacteriology*, 52 Int. J. Syst. Evol. Microbiol. 1043, 1045 (2002).

115 W. Ford Doolittle & R. Thane Papke, *Genomics and the Bacterial Species Problem*, 7 Genome  Biol. 116, 116.1 (2006) [hereinafter Doolittle & Papke].

116
*See* Parks *et al.*, *A Complete Domain-to-Species Taxonomy* at 1084.

117
*See* Donovan H. Parks *et al.*, *GTDB: an Ongoing Census of Bacterial and Archaeal Diversity Through a Phylogenetically Consistent, Rank Normalized and Complete Genome-Based Taxonomy*, 50 Nucleic  Acids  Res. D785, D792 (2022).

118 van Regenmortel & Mahy at 10.

119
*See* ICTV, *About Virus Taxonomic Classification*, International Committee on Taxonomy of Viruses, https://ictv.global/about/taxonomy (accessed Apr. 20, 2025).

120
*See generally Chakrabarty* (discussing the ‘444 Patent).

121
*See generally* Tan *et al.*; *see generally* Gilbert *et al.*

122
*See generally* Fang *et al*.

123
*See generally* Krammer *et al.*

124
*See generally* US Patent Application Serial No. 17/725,982.

125
*See generally* Jon C. Aster, *What Is in a Name? Consequences of the Classification Schism in Hematopathology*, 41 J. Clin. Oncol. 1523 (2023) [hereinafter Aster]; *see generally* Ignacio Sanz, *Challenges and Opportunities for Consistent Classification of Human B Cell and Plasma Cell Populations*, 10 Front. Immunol. 2458 (2019) [hereinafter Sanz *et al.*]; *see generally* Christina E. Zielinski, *T Helper Cell Subsets: Diversification of the Field*, 53 Eur. J. Immunol. e2250218 (2023) [hereinafter Zielinski *et al.*].

126
*See* Southwall Techs., Inc. v. Cardinal IG Co., 54 F.3d 1570, 1578 (Fed. Cir. 1995) (‘A patentee may not proffer an interpretation for the purposes of litigation that would alter the indisputable public record consisting of the claims, the specification and the prosecution history…’).

127 Immunex Corp. v. Sanofi-Aventis U.S. LLC, 977 F.3d 1212, 1221 (Fed. Cir. 2020).

128
*Id*.

129
*Id.*

130
*Id.* at 1216.

131 Eon Corp. IP Holdings LLC v. Silver Spring Networks, Inc., 815 F.3d 1314, 1320 (Fed. Cir. 2016) [citing to Phillips v. AWH Corp., 415 F.3d 1303, 1312, 1321 (Fed. Cir. 2005) (*en banc*)].

132
*See* Phillips, 415 F.3d at 1314–1319.

133
*Id.* at 1314; *see also* Middleton, Inc. v. Minn. Mining and Mfg. Co., 411 F.3d 1384, 1387 (Fed. Cir. 2002).

134 Phillips, 415 F.3d at 1315 (quoting Markman v. Westview Instruments, Inc., 52 F.3d 967, 978 (Fed. Cir. 1995), aff’d, 517 U.S. 370 (1996)).

135 Phillips, 415 F.3d at 1315.

136
*Id.* at 1316.

137
*Id.*

138
*Id.* at 1317.

139
*See id.* at 1318–1319.

140
*See id.* at 1317.

141 Sunovion Pharms., Inc. v. Teva Pharms. USA, Inc., 731 F.3d 1271 (Fed. Cir. 2013).

142
*Id.* at 1276.

143
*Id.* at 1277.

144 Genzyme, 346 F.3d 1094 at 1101.

145
*See id.* at 1101, 1103.

146
*See id.* at 1109 (Linn, J., concurring in part and dissenting in part).

147
*See* Genzyme, 346 F.3d at 1103–1104.

148 See Phillips, 415 F.3d at 1319.

149 Ma. Inst. of Tech. v. Abacus Soft., 462 F.3d 1344, 1351 (Fed. Cir. 2006).

150
*Id.* at 1351–1353.

151 Doolittle & Papke at 116.1.

152 Goris *et al.* at 81.

153
*See generally* Krammer *et al.*

154
*See generally* Aster; *see generally* Sanz *et al.*; *see generally* Zielinski *et al.*

155 Lindemann Maschinenfabrik GmbH v. American Hoist & Derrick Co., 730 F.2d 1452, 1463 (Fed. Cir. 1984).

156
*See generally* Chakrabarty (discussing the ‘444 Patent); US Patent No. 4,259,444, col. 3 l. 63 – col. 4 l. 12 (citing Stanier *et al.*).

157
*See generally* BASF (discussing *B. napus* and knowledge thereof in the prior art).

158
*See generally* Vaeck (discussing Cyanobacteria and knowledge thereof in the prior art).

159 Juno Therapeutics, Inc. v. Kite Pharma., 10 F.4th 1330, 1341 (Fed. Cir. 2021).

160 In re Buchner, 929 F.2d 660, 661 (Fed. Cir. 1991).

161
*See* National Center for Biotechnology Information, *Uncultured Clostridium sp. Isolate 2789STDY5608847, Whole Genome Shotgun Sequencing Project*, GenBank, https://www.ncbi.nlm.nih.gov/nuccore/FMEY00000000.1 (Aug. 16, 2016).

162
*See* Australian Centre for Ecogenomics, *GCA_900066195.1 Mediterraneibacter massiliensis 2789STDY5608847*, Genome Taxonomy Database, https://gtdb.ecogenomic.org/genome?gid=GCA_900066195.1#taxon-history (accessed May 28, 2025).

163 Thomas C. A. Hitch *et al.*, *Automated Analysis of Genomic Sequences Facilitates High-Throughput and Comprehensive Description of Bacteria*, 1 ISME Commun. 16, 16.11 (2021).

164
*See* Australian Centre for Ecogenomics, *Release 226*, Genome Taxonomy Database, https://data.ace.uq.edu.au/public/gtdb/data/releases/release226/226.0/auxillary_files/sp_clusters_r226.tsv (accessed Apr. 20, 2025).

165
*See* Australian Centre for Ecogenomics, *Taxon History*, Genome Taxonomy Database, https://gtdb.ecogenomic.org/taxon-history?from=R80&to=R226&query=g__Faecalicatena (accessed May 28, 2025).

166
*See generally* Amadou Hamidou Togo *et al.*, *Description of Mediterraneibacter massiliensis, gen. nov., sp. nov., a New Genus Isolated from the Gut Microbiota of an Obese Patient and Reclassification of Ruminococcus faecis, Ruminococcus lactaris, Ruminococcus torques, Ruminococcus gnavus and Clostridium glycyrrhizinilyticum as Mediterraneibacter faecis comb. nov., Mediterraneibacter lactaris comb. nov., Mediterraneibacter torques comb. nov., Mediterraneibacter gnavus comb. nov. and Mediterraneibacter glycyrrhizinilyticus comb. nov*, 111 Antonie  Van  Leeuwenhoek 2107 (2018).

167 Dominic Jarvis, *Scientists ‘Fix’ Bacterial Tree of Life*, The University of Queensland Australia, https://www.uq.edu.au/news/article/2018/08/scientists-fix-bacterial-tree-of-life (accessed Aug. 28, 2018).

168 Niall A. Logan, *Bacterial Systematics*, 173 (1994).

169 M.D. Collins *et al.*, *The Phylogeny of the Genus Clostridium: Proposal of Five New Genera and Eleven New Species Combinations*, 44 Int. J. Syst. Bacteriol. 812, 812 (1994).

170
*Id.*

171
*Eg* M.-C. Domingo *et al.*, *Clostridium lavalense sp. nov., a Glycopeptide-Resistant Species Isolated from Human Faeces*, 59 Int. J. Syst. Evol. Microbiol. 498, 498 (2009); Thomas Clavel *et al.*, *Clostridium saccharogumia sp. nov. and Lactonifactor longoviformis gen. Nov., sp. nov., Two Novel Human Faecal Bacteria Involved in the Conversion of the Dietary Phytoestrogen Secoisolariciresinol Diglucoside*, 30 Syst. Appl. Microbiol. 16, 16 (2007).

172
*See generally* Eieter Rössler *et al.*, *Phylogenetic Diversity in the Genus Bacillus as Seen by 16S rRNA Sequencing Studies*, 14 Syst. Appl. Microbiol. 266 (1991).

173 Parks *et al.*, *A Standardized Bacterial Taxonomy* at 997.

174
*See* Australian Centre for Ecogenomics, *Frequently Asked Questions*, Genome Taxonomy Database, https://gtdb.ecogenomic.org/faq#why-do-some-genus-and-species-names-end-with-an-alphabetic-suffix (accessed Apr. 20, 2025).

175
*See generally* Jason W. Sahl *et al.*, *Defining the Phylogenomics of Shigella Species: a Pathway to Diagnostics*, 53 J. Clin. Microbiol. 951 (2015).

176
*See generally* Michael Richter & Ramon Rosselló-Móra, *Shifting the Genomic Gold Standard for the Prokaryotic Species Definition*, 106 Proc. Natl. Acad. Sci. U S A 19126 (2009); *see generally* Ruiting Lan & Peter R. Reeves, *Escherichia coli in Disguise: Molecular Origins of Shigella*, 4 Microbes  Infect. 1125 (2002).

177 Donovan H. Parks *et al.*, *Reclassification of Shigella Species as Later Heterotypic Synonyms of Escherichia coli in the Genome Taxonomy Database*. 2021.09.22.461432 bioRxiv, 1 (2021).

178 DSMZ, *Genus Shigella*, List of Prokaryotic Names with Standing in Nomenclature, https://lpsn.dsmz.de/genus/shigella (accessed Apr. 13, 2025).

179
*See generally* Hay *et al.*, *The Evolution of Human Influenza Viruses*, 356 Philos. Trans. R Soc. Lond. B Biol. Sci. 1861 (2001).

180
*See generally* Krammer *et al.*

181
*See generally id.*

182
*See generally id.*

183 WHO.

184
*See generally* Krammer *et al.*

185 WHO.

186
*See generally* Allen & Ross.

187
*See* Jorquera *et al.* at Fig. 1.

188 222 F.3d 1347.

189
*Id.* at 1350.

190
*Id.* at 1352.

191
*Id.*

192 The Federal Circuit considered this amendment not to add new matter, finding that the inventor made no attempt to broaden the scope to cover polypeptides not disclosed at the time and reversed the district court’s finding on that issue. *Id.* at 1352–1353. Other jurisdictions with more stringent or formalistic approaches to added matter may not be so permissive, however.

193 Schering, 222 F.3d*.* at 1353.

194
*Id.* at 1353–1354.

195
*Id.* at 1354.

196
*See generally* Kopykake Enterprises, Inc. v. Lucks Co., 264 F.3d 1377, 1380 (Fed. Cir. 2001).

197
*Id.* at 1380.

198
*Id.* at 1383.

199
*Id.* at 1381.

200
*Id.* at 1383–1384.

201
*Id.* at 1384.

202 Johnson Johnston Assoc. v. R.E. Serv., 285 F.3d 1046 (Fed. Cir. 2002) (*en banc*).

203 Maxwell v. J. Baker, Inc., 86 F.3d 1098, 1106–1107 (Fed. Cir. 1996).

204 Johnson Johnston, 285 F.3d at 1054–1055.

205 YBM Magnex v. Int’l Trade Comm’n, 145 F.3d 1317 (Fed. Cir. 1998).

206 Johnson Johnston, 285 F.3d at 1055.

207 Intervet, 617 F.3d at 1285.

208
*Id.* at 1286.

209
*Id.* at 1287.

210 Àlex Olvera *et al.*, *Molecular Evolution of Porcine Circovirus Type 2 Genomes: Phylogeny and Clonality*, 357 Virology 175, 178 (2007) [hereinafter Olvera *et al.*].

211 Based on multiple alignment and pairwise comparisons of genomes published by Olvera *et al.* with Accession Nos. AY651850.1 (1A), AY847748.1 (1B), AY484410.1 (1C), AB072302.1 (2A), AY180397.1 (2B), AY256459.1 (2C), AY256458.1 (2D), and AY325495.1 (2E).

212
*See* Olvera *et al.* at 176.

213 Charles T. Parker *et al.*, *International Code of Nomenclature of Prokaryotes*, 69 Int. J. Syst. Evol. Microbiol. S1 (2019) [hereinafter ICNP], Principle 7.

214
*Id.*, Rules 15 and 27.

215
*Id.*, Rule 15.

216
*See* Xin Wang *et al.*, *A Novel Isolate of Clostridium butyricum for Efficient Butyric Acid Production by Xylose Fermentation*, 68 Ann. Microbiol. 321 (2018); *see* American Type Culture Collection, *Clostridium butyricum Prazmowski 19398™*, ATCC https://www.atcc.org/products/19398 (accessed Apr. 13, 2025) (describing ATCC 19398 (VPI 3266) as the type strain of *Clostridium butyricum*).

217 DSMZ, *Species Clostridium butyricum*, List of Prokaryotic Names with Standing in Nomenclature, https://lpsn.dsmz.de/species/clostridium-butyricum (accessed Apr. 13, 2025).

218
*See generally* C. S. Cummins & J. L. Johnson, *Taxonomy of the Clostridia: Wall Composition and DNA Homologies in Clostridium butyricum and Other Butyric Acid-producing Clostridia*, 67 J. Gen. Microbiol. 33 (1971) (comparing phenotypes and DNA homology with other strains); *see generally* Bo Xin, *Genome Sequence of Clostridium butyricum Strain DSM 10702, a Promising Producer of Biofuels and Biochemicals*, 1 Genome  Announc. e00563 (2013) (presenting a genome sequence).

219
*See generally* Katia Koelle *et al.*, *Epochal Evolution Shapes the Phylodynamics of Interpandemic Influenza A (H3N2) in Humans*, 314 Science 1898 (2006) [hereinafter Koelle *et al.*].

220
*See generally* Krammer *et al.*

221
*See generally id.*

222
*See generally id.*

223
*See generally id.*

224 In re Hogan, 559 F.2d 595, 605 (C.C.P.A. 1977).

225
*Id.*

226
*Id.* at 601.

227
*Id.* at 606.

228
*Id.* at 605.

229 Plant Genetic Sys., N.V. v. DeKalb Genetics Corp., 315 F.3d 1335 (Fed. Cir. 2003).

230
*Id.* at 1338.

231
*Id.*

232
*Id.*

233
*See id.* at 1337, 1340.

234
*Id.* at 1345.

235
*Id.* at 1345–1346.

236
*See,* eg Vas-Cath Inc. v. Mahurkar, 935 F.2d 1555, 1562–1563 (Fed. Cir. 1991).

237
*See* Juno, 10 F.4th at 1335.

238 In re Entresto, 125 F.4th 1090, 1094–1095 (Fed. Cir. 2025).

239
*Id.* at 1098–1099

240
*See* AbbVie Deutschland GmbH & Co., KG v. Janssen Biotech, Inc., 759 F.3d 1285, 1300 (Fed. Cir. 2014).

241
*Id.*

242
*Id.* at 1298–1300.

243
*Id.* at 1300.

244
*Id.*

245 Juno, 10 F.4th at 1330.

246
*Id.* at 1333.

247
*Id.* at 1339–1340.

248
*Id.* at 1339.

249
*See* Doolittle & Papke at 116.1; *see* van Regenmortel & Mahy at 10.

250
*See generally* Tomeu Viver *et al.*, *Towards Estimating the Number of Strains That Make up a Natural Bacterial Population*, 15 Nat. Commun. 544 (estimating the presence of 5500 to 11,000 genomovars of *Salinibacter ruber* in a single pond environment).

251
*See* Ludwig & Schleifer at 156.

252
*See generally* Parks *et al.*, *A Complete Domain-to-Species Taxonomy* (defining species clusters as genomes encompassed by a specified ANI to a representative genome).

253
*See generally* Koelle *et al.*

254
*See generally id.*; *see also* Huber *et al.*

255 See generally Koelle *et al.*

256
*See generally* Krammer *et al.*

257
*See* Philip Hugenholtz *et al.*, *Prokaryotic Taxonomy and Nomenclature in the Age of Big Sequence Data*, 15 ISME J. 1879, 1880 (2021).

258
*See generally* Parks *et al.*, *A Standardized Bacterial Taxonomy*.

259
*See generally* International Committee on Taxonomy of Viruses Executive Committee, *The New Scope of Virus Taxonomy: Partitioning the Virosphere into 15 Hierarchical Ranks*, 5 Nat. Microbiol. 668 (2020).

260 International Committee on Taxonomy of Viruses, *Genus: Betacoronavirus*, ICTV, https://ictv.global/report/chapter/coronaviridae/coronaviridae/betacoronavirus (accessed Apr. 13, 2025).

261
*See* Eugen Bauer *et al.*, *Phenotypic Differentiation of Gastrointestinal Microbes Is Reflected in Their Encoded Metabolic Repertoires*, 3 Microbiome 55, 6–7 (2015).

262
*See* Jennifer B. H. Martiny *et al.*, *Microbiomes in Light of Traits: a Phylogenetic Perspective*, 350 Science aac9323, 7 (2015) [hereinafter Martiny *et al.*].

263 Noel R. Krieg *et al.*, *Bergey’s Manual of Systematic Bacteriology: Volume 4: The Bacteroidetes, Spirochaetes, Tenericutes (Mollicutes), Acidobacteria, Fibrobacteres, Fusobacteria, Dictyoglomi, Gemmatimonadetes, Lentisphaerae, Verrucomicrobia, Chlamydiae, and Planctomycetes*, xi (2nd ed. 2010).

264 Martiny *et al.* at 7.

265
*See generally* Adam C. Martiny *et al.*, *Phylogenetic Conservatism of Functional Traits in Microorganisms*, 7 ISME J. 830 (2013) [hereinafter Martiny *et al.*, *Phylogenetic Conservatism*].

266 Joseph L. Sevigny *et al.*, *Marker Genes as Predictors of Shared Genomic Function*, 20 BMC Genomics 268, 5 (2019).

267
*See*, eg Cross v. Iizuka, 753 F.2d 1040, 1050 (Fed. Cir. 1985).

268
*See generally* Thorsten Kampmann *et al.*, *The Role of Histidine Residues in Low-pH-Mediated Viral Membrane Fusion*, 14 Structure 1481 (2006).

269
*See generally* Caroline M. Mair *et al.*, *A Histidine Residue of the Influenza Virus Hemagglutinin Controls the pH Dependence of the Conformational Change Mediating Membrane Fusion*, 88 J. Virol. 13189 (2014).

270
*See generally* Lauren Byrd-Leotis *et al.*, *Influenza Hemagglutinin (HA) Stem Region Mutations That Stabilize or Destabilize the Structure of Multiple HA Subtypes*, 89 J. Virol. 4504 (2015).

271
*See generally* Milder *et al*.

272
*See generally* Jarek Juraszek *et al.*, *Engineering a Cleaved, Prefusion-Stabilized Influenza B Virus Hemagglutinin by Identification and Locking of All Six pH Switches*, 3 PNAS Nexus pgae462 (2024).

273
*See generally Karshtedt et al.*

274 Regents of Univ. of Cal. v. Eli Lilly & Co., 119 F.3d 1559 (Fed. Cir. 1997).

275 Ariad Pharms., Inc. v. Eli Lilly & Co., 598 F.3d 1336 (Fed. Cir. 2010).

276 Juno, 10 F.4th at 1341.

277 In re Curtis, 354 F.3d 1347, 1356–1357 (Fed. Cir. 2004).

278 In re Smythe, 480 F.2d 1376, 1382–1383 (C.C.P.A. 1973).

279 Univ. Of Rochester v. G.D. Searle & Co., 358 F.3d 916 (Fed. Cir. 2004).

280 Union Oil Co. of California v. Atl. Richfield Co., 208 F.3d 989, 992 (Fed. Cir. 2000).

281
*Id.* at 1001.

282 Ajinomoto Co. v. Int’l Trade Comm’n, 932F.3d 1342, 1346 and 1359 (Fed. Cir. 2019).

283 BASF, 28 F.4th at 1253 (Fed. Cir. 2022).

284
*Id.* at 1265–1267.

285
*Id.* at 1266.

286
*Id.* at 1268–1269.

287
*Id.*

288 35 U.S.C. § 112(a).

289 Wands, 858 F.2d at 737.

290
*Id.*

291
*See generally* Lemley & Sherkow.

292
*See* Amgen v. Sanofi, 598 U.S. 594, 602 (2023); US Patent No. 8,829,165, col. 430 ll. 17–23.

293 Amgen, 598 U.S. at 614.

294
*Id.* at 611.

295 Baxalta Inc. v. Genentech, Inc., 81 F.4th 1362, 1363 (Fed. Cir. 2023).

296 Vaeck, 947 F.2d at 495–496.

297 Martek Biosciences Corp. v. Nutrinova, Inc., 579 F.3d 1363 (Fed. Cir. 2009).

298
*Id.* at 1378–1379.

299
*Id.* at 1379.

300 Amgen, 598 U.S. at 610.

301
*See*, eg Vas-Cath, 935 F.2d at 1562–1563.

302
*See* BASF, 28 F.4th at 1266.

303
*See* US Patent No. 9,926,579, col. 215 ll. 29–41 (reciting ‘A *Brassica* plant cell’).

304
*See* BASF, 28 F.4th at 1256, n. 1.

305
*See generally* Thomas J. Givnish, *Plant Biology: Phylogenomics of Mustards and Their Relatives*, 33 Curr. Biol. R998 (2023).

306
*See generally id.*

307
*See* Vaeck, 947 F.2d at 490–491.

308
*See id.*

309
*See* Martek Biosciences, 579 F.3d at 1378–1379.

